# The Role of Aldosterone in Vascular Permeability in Diabetes

**DOI:** 10.3390/cells15010089

**Published:** 2026-01-05

**Authors:** Michal Aleksiejczuk, Natalia Bielicka, Magdalena Bruzgo-Grzybko, Izabela Suwda Kalita, Adam Jan Olichwier, Paulina Mierzejewska, Joanna Stelmaszewska, Janusz Dzieciol, Ewa Chabielska, Anna Gromotowicz-Poplawska

**Affiliations:** 1 Department of Biopharmacy and Radiopharmacy, Medical University of Bialystok, 15-222 Bialystok, Poland; michal.aleksiejczuk@gmail.com (M.A.); natalia.bielicka@umb.edu.pl (N.B.); magdalena.bruzgo-grzybko@umb.edu.pl (M.B.-G.); ewa.chabielska@umb.edu.pl (E.C.); 2 Radiopharmacy Centre, Medical University of Bialystok, 15-569 Bialystok, Poland; izabela.kalita@umb.edu.pl (I.S.K.); adam.olichwier@umb.edu.pl (A.J.O.); 3 Department of Biochemistry, Medical University of Gdansk, 80-211 Gdansk, Poland; paulina.mierzejewska@gumed.edu.pl; 4 Department of Pharmaceutical and Biopharmaceutical Analysis, Medical University of Bialystok, 15-222 Bialystok, Poland; j.stelmaszewska@gmail.com; 5 Department of Human Anatomy, Medical University of Bialystok, 15-222 Bialystok, Poland; janusz.dzieciol@umb.edu.pl

**Keywords:** vascular permeability, aldosterone, eplerenone, vWF, VEGF, diabetes, mineralocorticoid receptor

## Abstract

More than 30% of diabetic patients develop dermatopathies linked to inflammation and increased vascular permeability. Considering the role of the renin–angiotensin–aldosterone system (RAAS) in diabetic complications, this study examined whether aldosterone (ALDO) and the mineralocorticoid receptor (MR) contribute to diabetes-related skin microangiopathy. Vascular permeability was measured in normoglycemic rats and insulin-dependent (streptozotocin-induced) diabetic rats. The expression of MR, 11β-hydroxysteroid dehydrogenase type 2 (HSD11β2), vascular endothelial growth factor (VEGF), von Willebrand factor (vWF), and the tight junction protein ZO-1 was determined by PCR and immunohistochemistry. Diabetic rats received the MR antagonist eplerenone (EPL, 100 mg/kg) for 10 days. Additionally, the effects of ALDO and EPL on endothelial permeability were evaluated in human dermal microvascular endothelial cells (HMEC-1) using a Transwell system. Diabetic rats showed skin atrophy, collagen damage, elevated ALDO levels, reduced MR and HSD11β2 expression, and increased vascular permeability, along with upregulation of VEGF and vWF. EPL markedly reduced these abnormalities. In vitro, ALDO increased endothelial permeability under hyperglycemia, and EPL counteracted this effect. These findings indicate that activation of the ALDO/MR pathway promotes skin vascular permeability in diabetes through VEGF- and vWF-dependent mechanisms. MR blockade limits these changes, suggesting therapeutic potential in preventing diabetes-associated skin complications.

## 1. Introduction

There is growing evidence that skin complications are associated with altered activity of the local aldosterone (ALDO)/mineralocorticoid receptor (MR) system, as well as changes in the expression of its components [1,2]. Given that extrarenal MR contributes to pathological processes in blood vessels, including inflammation, vasculopathy, and endothelial dysfunction, it is reasonable to suggest that the skin MR/ALDO system may play a similar role in inflammation-related skin disorders and vascular abnormalities [2,3]. Multiple mechanisms contribute to ALDO-induced endothelial dysfunction. These include impaired vascular tone, ALDO- and endothelium-dependent inflammation, and ALDO-associated atherosclerosis and vascular remodeling. MR activation by ALDO induces transcription of pro-inflammatory genes such as IL-1β, IL-6, CTLA-4, and PAI-1 [4,5]. ALDO can also enhance inflammation through non-genomic pathways [6]. Skin samples from patients with primary aldosteronism (PA) show epidermal hyperplasia, impaired differentiation, and increased dermal inflammatory infiltrates, changes associated with enhanced NF-κB signaling and elevated TNF-α and IL-6. These samples also display increased expression of MR, the glucocorticoid receptor (GR), and 11β-hydroxysteroid dehydrogenase type 2 (HSD11β2) [7]. Moreover, patients with PA exhibit reduced skin perfusion, negatively correlated with plasma ALDO levels, and greater microvascular dysfunction compared with individuals with essential hypertension [8]. Taken together, these findings support a pathophysiological role for ALDO in skin microcirculation.

Increased vascular permeability—which controls the exchange of substances between blood vessels, tissues, and organs—is closely tied to the integrity of the endothelium [9]. Inflammatory signals, shear stress, and growth factors such as VEGF and fibroblast growth factor can disrupt this barrier and increase vascular permeability, allowing larger molecules, including plasma proteins, to leak out of the vessels [10]. An experimental study reported that high-dose intravenous ALDO caused retinal edema in rats, suggesting that ALDO can also increase blood–retina barrier permeability [11].

Around 30% of diabetic patients experience skin complications. Serum ALDO levels rise several-fold in diabetic patients and in diabetic rat models [12,13], although its concentration in the skin under hyperglycemic conditions has not yet been measured. Irreversible nonenzymatic glycation of proteins, altered cellular redox balance, increased oxidative stress, and chronic inflammation contribute to endothelial dysfunction in diabetes [9,14]. In our earlier work, MR blockade with eplerenone (EPL) improved endothelium-dependent vascular function in both large and small arteries of STZ-induced diabetic rats [15].

We previously showed that acute intradermal ALDO application increased skin vascular permeability in normoglycemic rats, and that this effect was attenuated by EPL [16]. The present study therefore aimed to assess skin vascular permeability in an insulin-dependent (streptozotocin-induced) diabetic rat model. We examined the expression of MR and HSD11β2, which ensures MR selectivity for ALDO [17], as well as key inflammatory and permeability-related factors: vascular endothelial growth factor (VEGF) [10], von Willebrand factor (vWF) [18], and the junctional protein ZO-1 [19], using PCR and immunohistochemistry. In addition, we performed in vitro experiments to evaluate the effects of ALDO on endothelial permeability in human dermal microvascular endothelial cells (HMEC-1) under normal and high-glucose conditions.

## 2. Materials and Methods

### 2.1. Animals

All procedures involving animals were carried out in accordance with institutional guidelines and the EU Directive 2010/63/EU on the protection of animals used for scientific purposes, as well as the recommendations for the care and use of laboratory animals in biomedical research [20]. The study was approved by the Local Ethical Committee for Animal Testing at the Medical University of Bialystok (No. 4/2017 and No. 60/2021).

Wistar rats (Cmdb:Wi) were used in this study. The number of animals included in each experiment is shown in the figure legends. Because vascular leakage measured in the Miles assay can vary with age and body weight [21], all rats were selected to be similar with respect to both parameters. Animals were kept under a 12 h light/dark cycle with controlled temperature and humidity and had free access to standard chow and tap water. Food was withdrawn 24 h before the experiment, but water remained available.

To avoid variation related to daily fluctuations in serum aldosterone levels [22], all in vivo procedures were performed at the same time of day (9:00 a.m.). Rats were anesthetized with intraperitoneal pentobarbital sodium (45 mg/kg; Morbital, Biowet, Pulawy, Poland). After blood and tissue collection, euthanasia was completed by pentobarbital overdose followed by exsanguination.

### 2.2. Experimental Protocol

Diabetes was induced on day 0 with a single dose of streptozotocin (STZ; Sigma-Aldrich, St. Louis, MO, USA), as previously described [15]. This insulin-dependent (STZ-induced) diabetic rat model reflects type 1 diabetes. Briefly, rats received an intraperitoneal injection of STZ at 65 mg/kg, while control animals (NORM) were given an equivalent volume of citrate buffer. Diabetes was allowed to develop over 35 days. Blood glucose was measured using a OneTouch glucometer (CardioCheck, PTS Diagnostics, Indianapolis, IN, USA). Hyperglycemia was defined as a tail-vein glucose level above 200 mg/dL on day 3 and again on day 45 after STZ administration.

**Figure 1:**
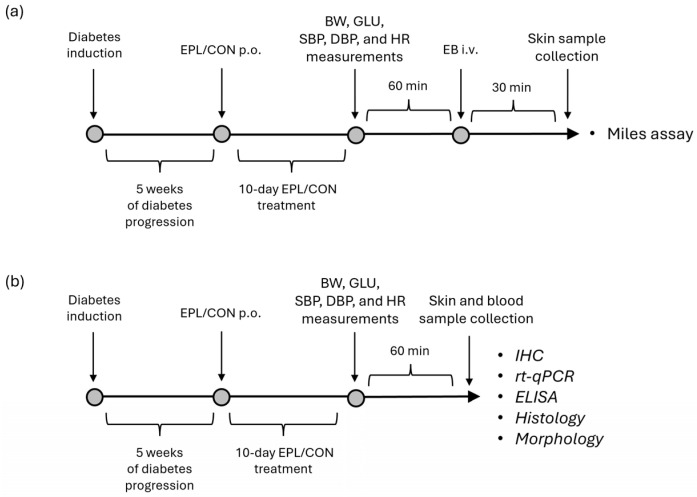
Schematic overview of the experimental design: (**a**) Miles assay–based vascular permeability assessment; (**b**) parallel histological and molecular analyses in a separate animal cohort. CON—eplerenone solvent; BW—body weight; DBP—diastolic blood pressure; EB—Evans blue; ELISA—enzyme-linked immunosorbent assay; EPL—eplerenone; GLU—glucose; HR—heart rate; IHC—immunohistochemistry; i.v.—intravenous; p.o.—per os; rt-qPCR—real-time quantitative polymerase chain reaction; SBP—systolic blood pressure.

On day 35, rats were treated with eplerenone (EPL; 100 mg/kg/day; Inspra, Pfizer, New York, NY, USA) in a 5% gum arabic solution or with the vehicle alone (CON), administered by intragastric gavage for 10 days. After the final EPL dose, animals were fasted overnight but had free access to water. On day 45, hemodynamic parameters, including systolic and diastolic blood pressure (SBP and DBP) and heart rate (HR), were measured using an invasive method, as previously described [23]. After the hemodynamic measurements, animals were divided into two groups. One group underwent the Miles assay using Evans blue (EB) dye (Figure 1a), whereas in the second group, histological, morphological, and immunohistochemical analyses were performed (Figure 1b). EB was administered to a separate group of animals, as EB staining of tissues would interfere with downstream analyses, particularly immunohistochemistry (IHC). Therefore, EB administration was not part of the experimental protocol used for RT-qPCR, IHC, ELISA, histological, or morphological analyses. A schematic overview of the experimental design is shown in Figure 1.

### 2.3. Vascular Permeability Measurement

Vascular permeability was assessed using the well-established Miles assay [24]. Rats were anesthetized, and Evans blue dye (EB; 30 mg/kg; Sigma-Aldrich, St. Louis, MO, USA) was subsequently administered via the femoral vein (Figure 1a). After 30 min, one skin sample (1.5 × 1.5 cm) from the dorsal region was collected from each rat, weighed, placed in formamide (4 mL), and incubated at 45 °C for 72 h. The samples were then centrifuged (2000 rpm for 30 min), and the dye concentration in the supernatant was measured colorimetrically at 620 nm. Results are expressed as EB content per gram of skin tissue.

### 2.4. Histological and Immunohistochemical Analysis

The rats were anesthetized, and skin biopsies were collected from the dorsal region and then fixed in 4% phosphate-buffered formaldehyde and embedded in paraffin. Sections 4 µm thick were cut and stained with hematoxylin and eosin (H&E) for general histological assessment. An experienced histologist, blinded to the experimental groups, evaluated the samples using an OLYMPUS imaging system (BX50 microscope, DP20 camera, CellD software ver. 3.1; OLYMPUS, Tokyo, Japan). To assess structural changes, the thickness of the epidermis, dermis, and hypodermis was measured and expressed as a percentage of the total skin thickness.

Paraffin-embedded sections were used for immunohistochemical (IHC) staining. IHC analysis was performed to assess the protein expression of vWF, VEGF, MR, HSD11β2, and ZO-1, factors potentially involved in regulating vascular permeability. All procedures were carried out using commercially available kits, following the manufacturers’ instructions. The following primary antibodies were used: vWF (kit No. M0616; DakoCytomation, Glostrup, Denmark), VEGF (kit No. M7273; DakoCytomation, Denmark), MR (kit No. ab2774; Abcam, Cambridge, UK), HSD11β2 (kit No. ab203132; Abcam, UK), and ZO-1 (kit No. 214228; Abcam, UK).

A positive reaction was indicated by brown staining corresponding to antigen–antibody complexes at the site of the target protein. Staining intensity was graded on a scale from (−) to (+++), where (−) indicated no detectable signal, (+) a weak signal, (++) a moderate signal, and (+++) a strong signal. Positively stained cells were examined under high-power fields using the same OLYMPUS imaging system.

### 2.5. mRNA Extraction and Real-Time Quantitative rt-PCR

To assess the mRNA expression of vWF, VEGF, MR, HSD11β2, and ZO-1, real-time quantitative PCR (rt-qPCR) was performed as previously described [16,25]. Gene expression levels were normalized to the housekeeping gene β-actin (Actb). Relative expression was calculated using the qBase MS Excel VBA applet. The primer sequences (5′→3′) were:

- VEGF: F: CATCAGCCAGGGAGTCTGTG; R: GAGGGAGTGAAGGAGCAACC
- vWF: F: GCTCAGGGACATGGCTTAGG; R: CCATACAAACAGGGGCCGTA
- MR: F: CCATGCAGGCAACATTACCG; R: GTAAGAAAGGCCCCACCCTC
- HSD11β2: F: CCAGCCACATGGAAGCTGTA; R: CAAACACTATCTCTCCCATTCTAGG
- ZO-1: F: GGTAGTGCAAAGAGATGAGC; R: GGCATTAGCAGAATGGATAC
- Actb: F: GCAGGAGTACGATGAGTCCG; R: ACGCAGCTCAGTAACAGTCC

### 2.6. Blood Morphology

Blood morphology was assessed using a Scil-Vet ABC Plus+ hematological analyzer (Horiba ABX, Montpellier, France). Cell counting was performed using the volumetric impedance method. The analyzer provided direct measurements of white blood cells (WBC), red blood cells (RBC), hemoglobin (HGB), and platelet count (PLT), along with automatic calculation of hematocrit (HCT).

### 2.7. Measurement of Blood Glucose Concentration

Twenty-four hours before blood glucose measurement, the animals were deprived of food but had unlimited access to water. The animals were restrained using a restrainer, and blood was collected by puncturing the tail vein. Glucose levels were measured with a commercially available Optium Xido glucometer (Abbott Diabetes Care Inc., Alameda, CA, USA) and its dedicated test strips, following the manufacturer’s instructions.

### 2.8. Serum and Skin ALDO Concentration

The serum and skin levels of ALDO were measured using an ELISA kit (No. 501090; Cayman Chemical, Ann Arbor, MI, USA). Serum ALDO concentrations were determined according to the manufacturer’s instructions. Skin ALDO levels were assessed using a modified version of the method described by Reynoso-Palomar et al. [26]. Skin fragments were homogenized in phosphate-buffered saline (1 mL per 100 mg of tissue). To disrupt cell membranes, two freeze–thaw cycles were performed. Chloroform extraction was then carried out following the ELISA kit protocol. After chloroform evaporation, the residue was dissolved in ELISA buffer, and ALDO concentrations were measured according to the manufacturer’s instructions.

### 2.9. Cell Cultures

Immortalized human dermal microvascular endothelial cells (HMEC-1; ATCC^®^ CRL-3243™; ATCC, Glasgow, UK) were maintained at 37 °C in a 5% CO_2_ atmosphere in MCB131 medium (Gibco™, Thermo Fisher Scientific, Waltham, MA, USA) supplemented with 10% fetal bovine serum, streptomycin–penicillin (100 U/mL; Sigma-Aldrich, USA), L-glutamine (2 mM; Sigma-Aldrich, USA), hydrocortisone (1 μg/mL; Sigma-Aldrich, USA), heparin (25 U; Sigma-Aldrich, USA), and endothelial growth factor (30 μg/mL; Sigma-Aldrich, USA). Cells were used for no more than four passages.

HMEC-1 cells were seeded onto the upper surface of Transwell PET membrane inserts (1 µm pore size; #353097; Corning Inc., Corning, NY, USA) placed in a 24-well companion plate (#353504; Corning Inc., USA). After 24 h, a confluent monolayer was obtained. Nonadherent cells were removed, and the medium was replaced with either normoglycemic (NORM; 5.5 mM glucose + 24.5 mM mannitol) or hyperglycemic (HG; 30 mM glucose) medium, in which the cells were incubated for an additional 24 h.

ALDO (10^−10^–10^−7^ M) was added to NORM or HG culture medium and incubated for 15 or 60 min. The apical chambers were then placed into basal chambers filled with medium without FBS. A bovine serum albumin–Evans blue solution (BSA-EB; 0.5% Evans blue in PBS with 0.1% BSA) was added to the apical chamber, and the plates containing the Boyden chambers were incubated for 30 min in a CO_2_ incubator. Absorbance was measured spectrophotometrically at 610 nm (Synergy HT ST-00484, Biotek Instruments, Winooski, VT, USA). HMEC-1 permeability was defined by the absorbance value. Medium without Evans blue served as the reference, with transmittance defined as 100%.

EPL was added to the NORM or HG culture medium (final concentration 10^−5^ M; manufacturer) or an equivalent volume of culture medium was added for the control (CON), and the cells were incubated for 30 min. After this period, ALDO was added to a final concentration of 10^−9^ M and the cells were incubated for an additional 15 or 60 min. The apical chambers were then placed into the basal chambers (filled with FBS-free medium). BSA-EB solution was added to the apical chamber, and the plates containing the Boyden chambers were incubated for 30 min in a CO_2_ incubator. After incubation, absorbance was measured spectrophotometrically at 610 nm.

### 2.10. Statistical Analysis

Data are presented as mean ± SD. Normality was assessed using the Shapiro–Wilk test (and inspection of residual plots where applicable). Homogeneity of variances was evaluated using the Brown–Forsythe test.

For primary experimental outcomes with a factorial design, statistical analyses were performed using two-way analysis of variance (ANOVA) with disease status and treatment as independent factors, allowing assessment of main effects and their interaction. When appropriate, post hoc multiple comparisons were conducted using Tukey’s test. Statistical relationships among groups were visualized using compact letter displays, where groups sharing the same letter are not significantly different, whereas groups without a common letter differ significantly (*p* < 0.05).

For analyses not involving a factorial structure, or in cases where assumptions of homogeneity of variances were violated, group differences were evaluated using Welch’s one-way ANOVA followed by Tamhane’s T2 multiple comparisons test.

All statistical analyses were performed using GraphPad Prism v. 10.3.1 (GraphPad Software, San Diego, CA, USA).

## 3. Results

### 3.1. General Characteristic of Rats

In the STZ group, glycemia was markedly elevated compared with NORM animals (NORM+CON vs. STZ+CON: 65 ± 60 mg/dL vs. 285 ± 188 mg/dL; *p* < 0.001). A significant reduction in body weight was also observed (371 ± 52 g vs. 201 ± 40 g; *p* < 0.001). EPL treatment did not affect either blood glucose or body weight in any group. The values of SBP, DBP, and HR were similar across all groups and were not altered by EPL (Table S1). STZ rats showed a significant decrease in platelet count (PLT; *p* < 0.01) and WBC (*p* < 0.05) compared with NORM controls. EPL did not alter any hematological parameters in either NORM or STZ animals (Table S2).

Induction of diabetes increased mortality. In the NORM group, the survival rate was 100%, whereas in the STZ+CON group it was 80%. In the STZ+EPL group, survival increased to 90%. STZ rats were noticeably less active, displayed depressive behavior, reacted poorly to external stimuli, and neglected grooming; increased thirst and polyuria were also evident. EPL administration improved overall activity in STZ rats, and the animals resumed social interactions and grooming. As expected from the diuretic properties of EPL, both NORM and STZ rats showed increased thirst and diuresis.

**Figure 2:**
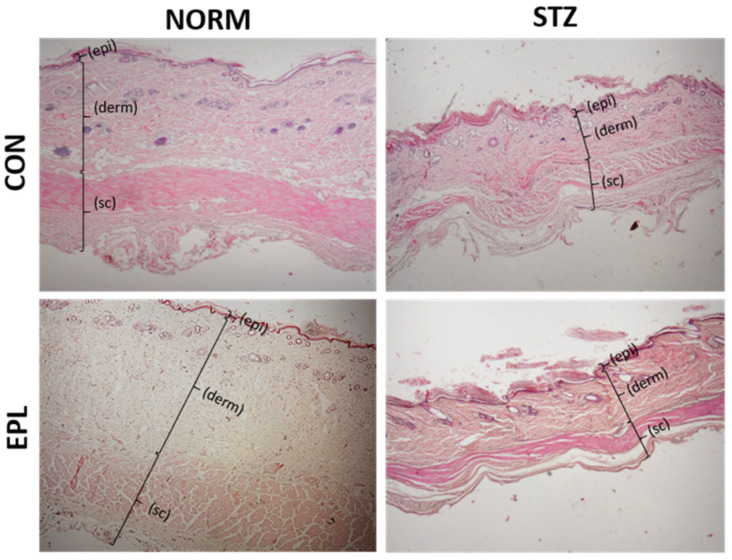
Effect of diabetes and/or eplerenone administration on the microscopic image of skin cross-sections with H+E staining. CON—eplerenone solvent; EPL—eplerenone; NORM—normoglycemic group; STZ—diabetic group; (epi)—epidermis; (derm)—dermis; (sc)—subcutaneous tissue; H+E—hematoxylin and eosin. Magnification ×100.

Histologically, skin from NORM rats displayed normal architecture: a clearly defined epidermis, well-organized dermal collagen with visible skin appendages, panniculus carnosus, and adipose lobules in the subcutaneous layer. In STZ rats, the epidermis retained normal structure, but the dermis exhibited pronounced atrophy with areas of collagen degeneration (Figure S1). The dermal muscle layer showed reduced thickness and density. A marked atrophy of subcutaneous tissue and significant loss of fat were also observed. Additionally, an increased number of monocytes was present in skin sections from STZ rats. These alterations significantly disrupted the proportional thickness of individual skin layers. EPL did not modify the histological appearance of the skin in either NORM or STZ groups (Figure 2, Table 1).

### 3.2. Vascular Permeability

**Figure 3:**
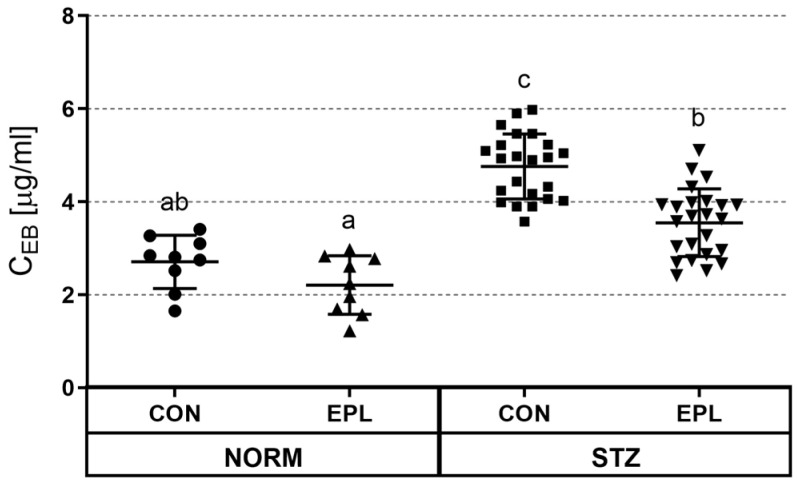
Effect of diabetes and/or eplerenone (EPL) administration on rat skin vascular permeability. C_EB_—Evans blue concentration; CON—eplerenone solvent; EPL—eplerenone; NORM—normoglycemic group; STZ—diabetic group. The results are presented as the mean ± SD; n = 9–23. Statistical relationships among groups were visualized using compact letter displays, where groups sharing the same letter are not significantly different, whereas groups without a common letter differ significantly (*p* < 0.05).

In the STZ group, skin vascular permeability was significantly increased compared with the NORM group (NORM+CON vs. STZ+CON: 2.71 ± 0.57 μg/mL vs. 4.76 ± 0.70 μg/mL; *p* < 0.001) (Figure 3). In NORM rats, EPL administration tended to reduce vascular permeability; however, this change did not reach statistical significance (NORM+CON vs. NORM+EPL: 2.71 ± 0.57 μg/mL vs. 2.21 ± 0.63 μg/mL). In contrast, in STZ rats, EPL treatment markedly and significantly reduced vascular permeability (STZ+CON vs. STZ+EPL: 4.76 ± 0.70 μg/mL vs. 3.55 ± 0.73 μg/mL; *p* < 0.001).

### 3.3. ALDO Concentration in Serum and Skin Homogenates

**Figure 4:**
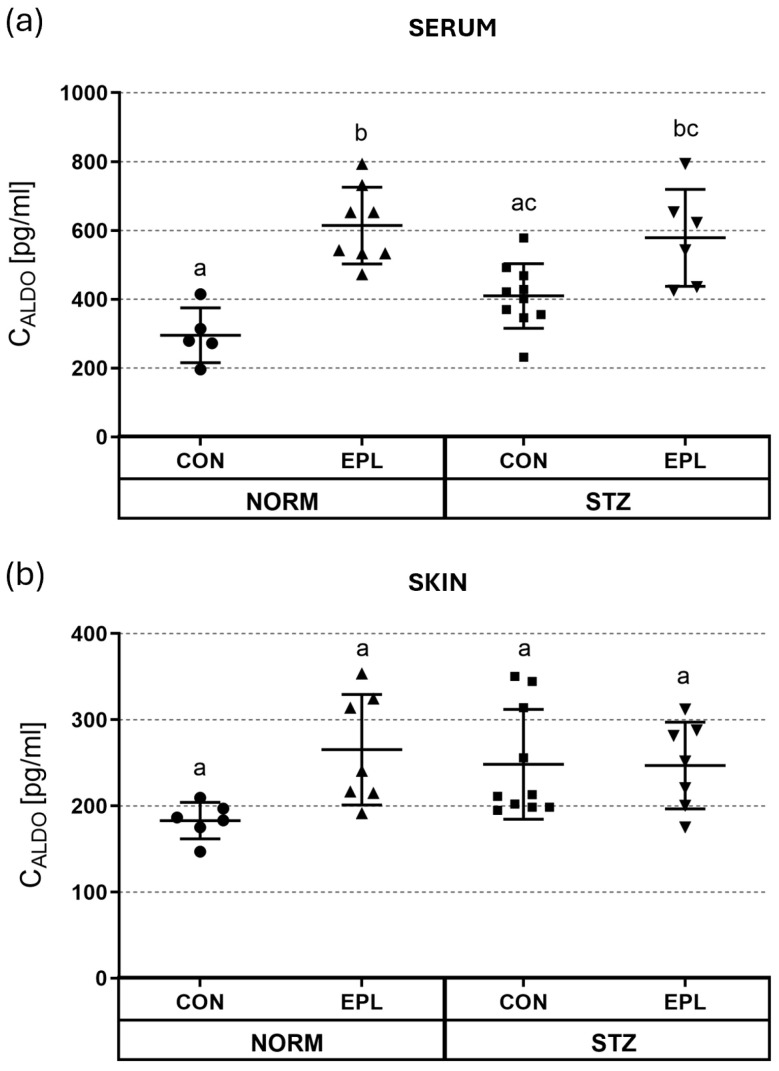
Effect of diabetes and/or administration of eplerenone (EPL) on (**a**) serum; (**b**) skin aldosterone (ALDO) levels. C_ALDO_—aldosterone concentration; CON—eplerenone solvent; EPL—eplerenone; NORM—normoglycemic group; STZ—diabetic group. Results are presented as mean ± SD; n = 5–10. Statistical relationships among groups were visualized using compact letter displays, where groups sharing the same letter are not significantly different, whereas groups without a common letter differ significantly (*p* < 0.05).

In the STZ group, serum ALDO concentration was not significantly different from that in the NORM group (NORM+CON vs. STZ+CON: 295.2 ± 79.5 pg/mL vs. 409.6 ± 94.1 pg/mL) (Figure 4a). In NORM rats, EPL administration increased serum ALDO levels by approximately 100% (NORM+CON vs. NORM+EPL: 295.2 ± 79.5 pg/mL vs. 614.3 ± 111.6 pg/mL; *p* < 0.01). In the STZ group, EPL administration did not significantly change serum ALDO levels (STZ+CON vs. STZ+EPL: 409.6 ± 94.1 pg/mL vs. 578.8 ± 140.6 pg/mL).

No significant differences in skin ALDO concentrations were observed among the studied groups. Neither the comparison between NORM and STZ rats nor the administration of EPL resulted in statistically significant changes in skin ALDO levels (Figure 4b).

### 3.4. Immunohistochemical Findings for MR, HSD11β2, VEGF, vWF, and ZO-1 in Skin

In whole-skin sections stained for MR, the signal was detected in the epidermis, blood vessels, hair follicles, and sebaceous glands. In NORM rats, staining intensity was moderate (++). In STZ rats, the staining was clearly weaker (+), indicating that diabetes reduced MR staining intensity relative to the NORM group (Figure S2). EPL administration did not alter the staining pattern in either NORM or STZ rats.

**Figure 5:**
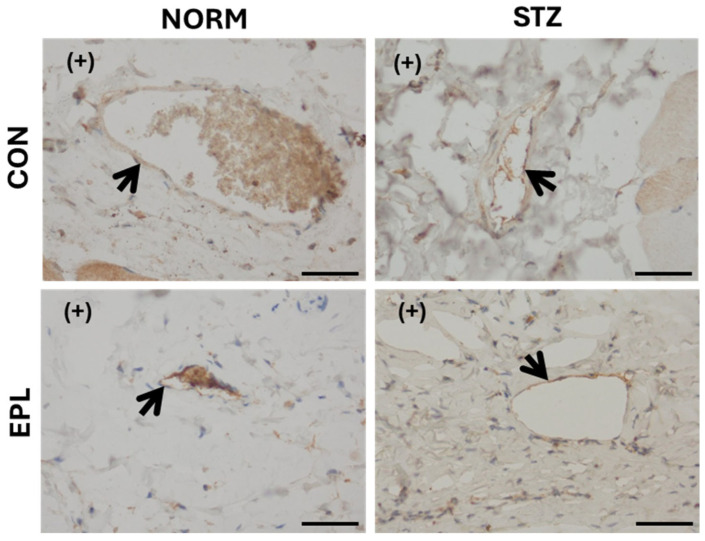
Microscopic image of skin blood vessels with immunohistochemical staining for the mineralocorticoid receptor (MR). A positive antigen–antibody reaction appears as a tan-brown color. In all groups, only weak staining was observed. Blood vessel walls are indicated by arrows. NORM—normoglycemic group; CON—eplerenone solvent; EPL—eplerenone; STZ—diabetic group; (+)—poor staining. Magnification ×400. Scale bars: 50 μm.

In microscopic images of skin blood vessels stained for MR, a weak (+) signal was observed in both NORM and STZ groups. EPL treatment did not alter staining intensity in either group (Figure 5).

Microscopic evaluation of full-thickness skin sections stained for HSD11β2 showed a positive signal in the epidermis, blood vessels, hair follicles, and sebaceous glands. In NORM rats, staining intensity was moderate (++). In the STZ group, staining was weaker (+), indicating that diabetes reduced HSD11β2 staining in the skin (Figure S3). EPL treatment did not affect staining intensity in either NORM or STZ rats.

**Figure 6:**
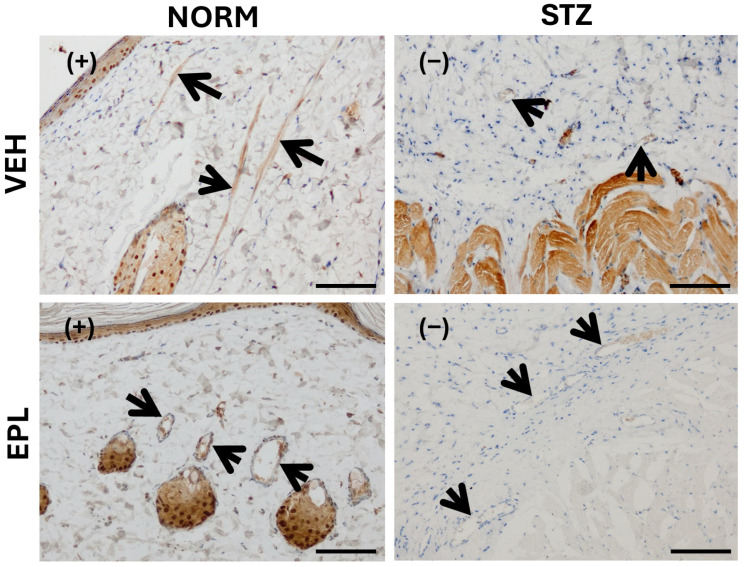
Effect of diabetes and/or eplerenone (EPL) administration on the microscopic image of skin blood vessels with immunohistochemical staining against 11β hydroxysteroid dehydrogenase (HSD11β2). A positive antigen–antibody reaction is seen as a tan-brown color. In the studied groups, a color reaction of weak intensity (+) was observed in the NORM group. There was no color reaction in the STZ (−) group. EPL did not affect the intensity of the color reaction. The walls of blood vessels are marked with an arrow. CON—eplerenone solvent; EPL—eplerenone; NORM—normoglycemic group; STZ—diabetic group. (+)—weak color reaction (−)—no color reaction. Magnification ×400. Scale bars: 50 μm.

Microscopic images of skin blood vessels stained for HSD11β2 showed weak (+) staining in the NORM group and no detectable staining (−) in the STZ group. EPL treatment did not alter staining intensity in either NORM or STZ rats (Figure 6).

In microscopic images of full-thickness skin sections stained for VEGF, staining was observed only within the blood vessels (Figure S4). Because no staining was detected in other skin structures, overall staining intensity for the full skin section was classified as absent (−). Diabetes and/or EPL treatment did not affect the staining intensity.

**Figure 7:**
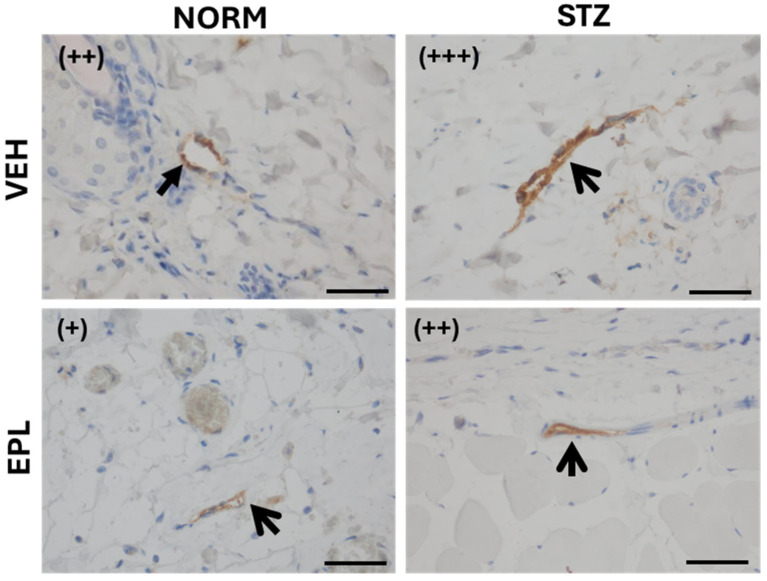
Effect of diabetes and/or eplerenone (EPL) treatment on the microscopic appearance of skin blood vessels stained immunohistochemically for vascular endothelial growth factor (VEGF). A positive antigen–antibody reaction appears as a tan-brown color. Moderate staining (++) was observed in the NORM group, while strong staining (+++) was present in the STZ group. EPL treatment reduced staining intensity to (+) in the NORM group and to (++) in the STZ group. CON—eplerenone solvent; EPL—eplerenone; NORM—normoglycemic group; STZ—diabetic group. (+)—poor staining; (++)—moderate staining; (+++)—strong staining. Magnification ×400. Scale bars: 50 μm.

Microscopic images of skin blood vessels stained for VEGF showed moderate staining (++) in the NORM group and strong staining (+++) in the STZ group. Following EPL treatment, staining intensity decreased to weak (+) in the NORM group and to moderate (++) in the STZ group (Figure 7).

In microscopic images of full-thickness skin sections stained for vWF, staining was observed only within the blood vessels (Figure S5). Because no staining was detected in other skin structures, the overall staining intensity for the full skin section was classified as absent (−). Diabetes and/or EPL treatment did not affect staining intensity.

**Figure 8:**
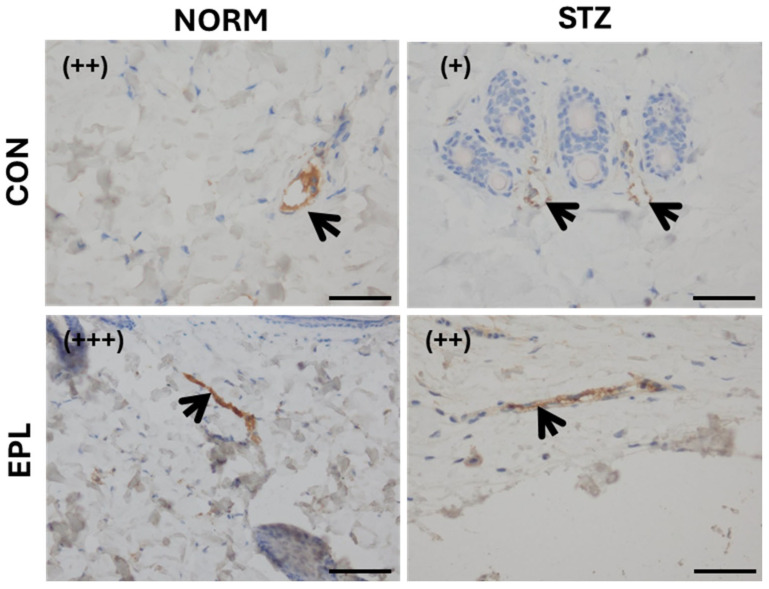
Effect of diabetes mellitus and/or eplerenone (EPL) administration on the microscopic picture of vessels immunohistochemical staining for von Willebrand factor (vWF). A positive antigen–antibody reaction is seen as a tan-brown color. A moderate staining (++) was observed in the NORM group and a weak staining (+) in the STZ group. EPL administration increased the intensity of staining in the NORM group to (+++) and in the STZ group to (++). CON—eplerenone solvent; EPL—eplerenone; NORM—normoglycemic group; STZ—diabetic group. (+)—poor staining; (++)—moderate staining; (+++)—strong staining. Magnification ×400. Scale bars: 50 μm.

In microscopic images of skin blood vessels stained for vWF, moderate staining (++) was observed in the NORM group, while staining intensity was weak (+) in the STZ group. Following EPL treatment, staining intensity increased, showing strong staining (+++) in the NORM group and moderate staining (++) in the STZ group (Figure 8).

In microscopic images of full-thickness skin sections stained for ZO-1, weak staining (+) was observed in the epidermis, hair follicles, sebaceous glands, and blood vessels. Diabetes and/or EPL treatment did not influence staining intensity (Figure S6).

**Figure 9:**
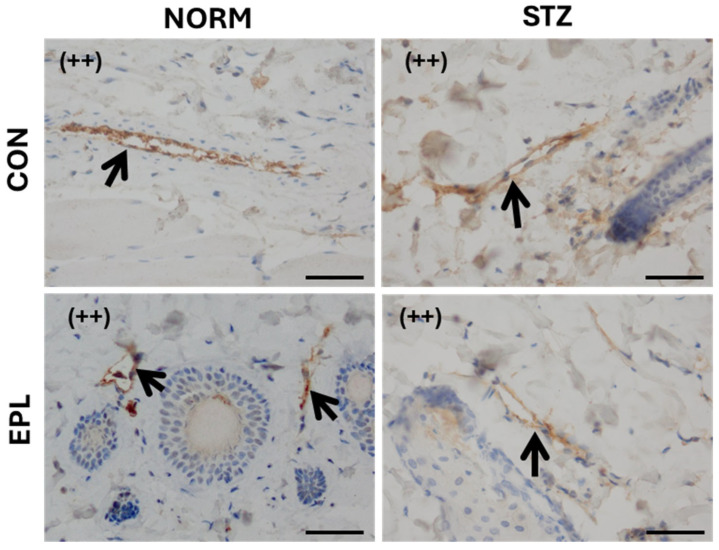
Effect of diabetes and/or eplerenone (EPL) administration on the microscopic image of skin blood vessels with immunohistochemical staining for the zonula occludens 1 (ZO-1). A positive antigen–antibody reaction is seen as a tan-brown color. A moderate staining (++) was observed in the NORM and STZ groups. EPL application did not affect the intensity of staining. CON—eplerenone solvent; EPL—eplerenone; NORM—normoglycemic group; STZ—diabetic group. (++)—moderate color reaction. Magnification ×400. Scale bars: 50 μm.

In microscopic images of skin blood vessels stained for ZO-1, moderate staining (++) was observed in both the NORM and STZ groups. EPL treatment did not alter staining intensity in either group (Figure 9).

### 3.5. mRNA Level of MR, HSD11β2, vWF, VEGF, and ZO-1

**Figure 10:**
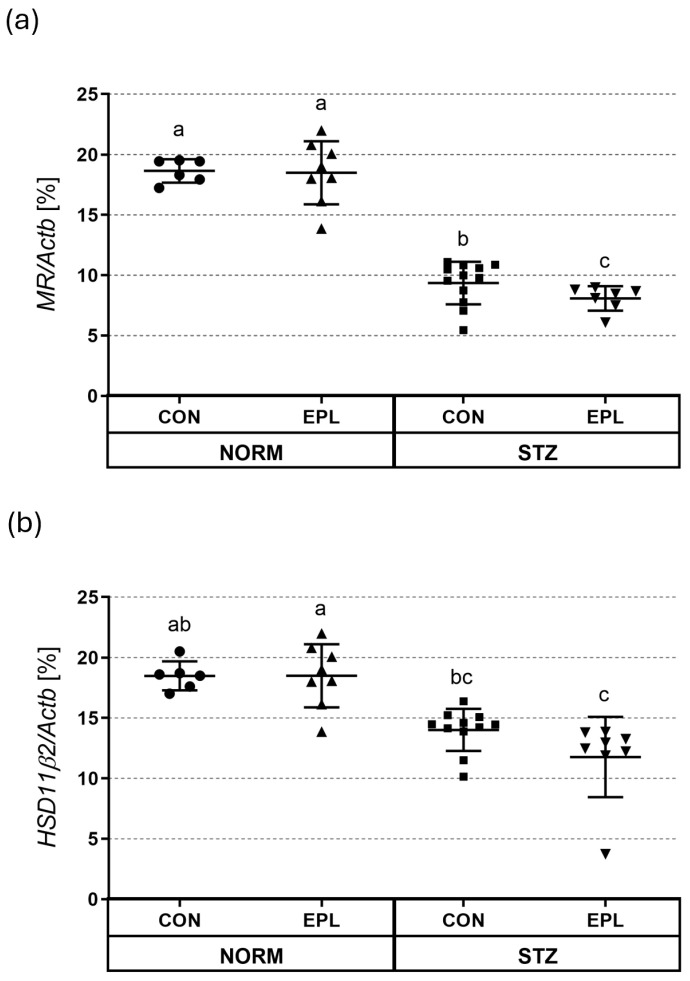
Effect of diabetes and/or eplerenone (EPL) administration on (**a**) mineralocorticoid receptor (MR); (**b**) 11β-hydroxysteroid dehydrogenase (11HSDβ2) mRNA expression in the skin. Actb—β-actin; CON—eplerenone solvent; EPL—eplerenone; NORM—normoglycemic group; STZ—diabetic group. Results are presented as mean ± SD; n = 6–12. Statistical relationships among groups were visualized using compact letter displays, where groups sharing the same letter are not significantly different, whereas groups without a common letter differ significantly (*p* < 0.05).

In all skin homogenates, mRNA expression of MR, HSD11β2, vWF, VEGF, and ZO-1 was detected. In the STZ group, MR expression in skin homogenates was significantly reduced compared with NORM rats (NORM+CON vs. STZ+CON; 18.64 ± 0.97 vs. 9.35 ± 1.76; *p* < 0.001). EPL treatment did not affect MR expression in NORM (NORM+CON vs. NORM+EPL; 18.64 ± 0.97 vs. 18.49 ± 2.61), but significantly reduced MR expression in STZ rats (STZ+CON vs. STZ+EPL; 9.35 ± 1.76 vs. 8.08 ± 1.01; *p* < 0.05) (Figure 10a).

HSD11β2 expression in skin homogenates showed a tendency toward lower values in STZ rats compared with normoglycemic controls; however, this difference did not reach statistical significance (NORM+CON vs. STZ+CON; 18.48 ± 1.19 vs. 13.36 ± 1.74; *p* = 0.083). EPL treatment did not affect HSD11β2 expression in either NORM (NORM+CON vs. NORM+EPL; 18.48 ± 1.19 vs. 18.35 ± 2.26) or STZ rats (STZ+CON vs. STZ+EPL; 13.36 ± 1.74 vs. 11.77 ± 3.32) (Figure 10b). In contrast, significant differences were detected in cross-comparisons involving EPL-treated groups, with HSD11β2 expression being significantly lower in STZ+EPL compared with both NORM+CON and NORM+EPL groups (*p* < 0.01 and *p* < 0.001, respectively) (Figure 10b).

No differences in the expression of vWF, VEGF, or ZO-1 were noted between the groups (Table 2).

### 3.6. In Vitro Study

#### 3.6.1. The “Effect-Dose” Parameter for ALDO

Preliminary tests showed that ALDO produced the strongest effect on paracellular permeability after 15 and 60 min at a concentration of 10^−9^ M (Figure S7). After 15 min of ALDO exposure, a significant increase in permeability was observed only at 10^−9^ M ALDO under both NORM (NORM+VEH vs. NORM+ALDO 15 min 10^−9^ M: 100 ± 2.6 vs. 111.1 ± 4.3; *p* < 0.05) and HG conditions (HG+VEH vs. HG+ALDO 15 min 10^−9^ M: 108.2 ± 3.8 vs. 123.5 ± 6.1; *p* < 0.05). With 60 min of ALDO exposure, a significant increase in permeability was found under NORM conditions at ALDO concentrations of 10^−9^ M and 10^−7^ M (*p* < 0.01 and *p* < 0.05, respectively). No differences in basal permeability were observed between cells incubated under NORM and HG conditions.

#### 3.6.2. The Effect of EPL on the ALDO-Increased Cell Permeability

**Figure 11:**
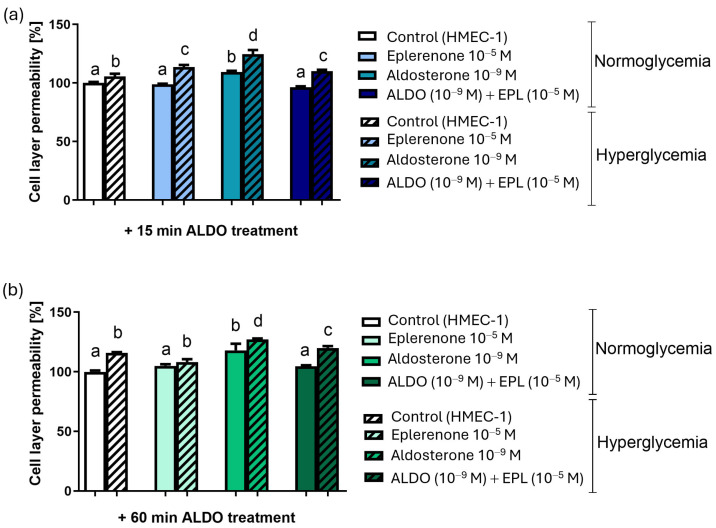
Permeability of the human dermal microvascular endothelial cell (HMEC-1) monolayer under normal (NORM) or hyperglycemic (HG) conditions after MR blockade; (**a**) with 15 min of aldosterone exposure, and (**b**) with 60 min of aldosterone exposure. ALDO—aldosterone; EPL—eplerenone. Results are presented as mean ± SD; n = 6–9. Statistical relationships among groups were visualized using compact letter displays, where groups sharing the same letter are not significantly different, whereas groups without a common letter differ significantly (*p* < 0.05).

To assess the contribution of the MR to the ALDO-increased permeability, cells were preincubated with EPL (10^−5^ M) for 30 min. EPL completely abolished the effect of ALDO 10^−9^ M after 15 min of exposure under both NORM (*p* < 0.001) and HG (*p* < 0.05) conditions. Permeability changes induced by 60 min of ALDO incubation were also reduced by EPL, under both NORM (*p* < 0.05) and HG (*p* < 0.05) conditions. EPL alone did not alter permeability in any experimental group (Figure 11).

## 4. Discussion

This is the first in vivo study to provide direct evidence that the ALDO/MR system contributes to vascular permeability in the skin microvasculature of an insulin-dependent (STZ-induced) diabetic rat model. Our findings expand on earlier work by demonstrating the involvement of the ALDO/MR system in regulating skin vessel permeability.

The findings also provide additional methodological insight into the STZ-induced model of diabetes, enriching the understanding of diabetes-induced alterations in skin structure and function. Five weeks after STZ administration, typical clinical features of diabetes—weight loss, polyuria, polydipsia, and elevated blood glucose—were observed. Histological evaluation confirmed the characteristic atrophic changes reported in this model, including thinning of the epidermis, collagen degradation, disruption of the dermal layer, and atrophy or loss of subcutaneous fat [27,28,29]. An increased number of inflammatory cells further indicated ongoing inflammation in the skin. No structural alterations were observed in the skin of STZ rats after 10 days of EPL treatment. EPL was administered at a dose of 100 mg/kg, selected based on our previous studies and published pharmacokinetic data in rats [15,23,30]. This dose effectively blocks aldosterone-dependent vascular effects without affecting systemic hemodynamic parameters.

It is well established that ALDO is produced locally in the skin [16,31,32,33]; however, this is the first study evaluating the skin MR/ALDO system in diabetes. In our study, MR expression in skin vessels did not differ between STZ and NORM rats. Data regarding MR expression changes in diabetes are scarce; one in vitro study showed that exposing human renal glomerular endothelial cells (HRGECs) to high glucose and angiotensin II (Ang II) did not alter MR expression [34]. MR expression has been reported in both mouse and human skin, particularly in epidermal keratinocytes and skin appendages such as hair follicles, sebaceous glands, and sweat glands, as well as in the skin of healthy rats, including vascular endothelial cells and nociceptive neurons [3,16,35].

EPL treatment did not affect MR expression in skin vessels in either NORM or STZ rats. However, we observed a marked reduction in MR expression in hair follicles and sebaceous glands of STZ rats, and a slight decrease in the epidermis. MR expression was also reduced in skin homogenates from STZ animals. In contrast, Nguyen et al. reported a sixfold increase in MR mRNA expression in skin homogenates of STZ mice [36]. Increased MR expression has been reported in the retina of Goto–Kakizaki rats (a type 2 diabetes model) and in humans [37]. Similarly, increased MR expression has been found in the renal cortex of mice [38] and in STZ rats and db/db mice [39]. Taken together, these findings suggest that changes in MR expression during diabetes are local and highly tissue- or structure-specific.

In our study, EPL had no effect on MR expression in any of the skin structures examined. There are no published data on how MR blockade influences MR expression in the skin in diabetes. However, in the kidneys of db/db mice and STZ rats, EPL reduced the diabetes-related increase in MR expression [39].

MR and GR belong to the same NR3C nuclear receptor subfamily and can both be activated by mineralocorticoids and glucocorticoids. MR is not selective for ALDO; circulating cortisol (CORT) concentrations are up to 1000-fold higher than ALDO levels. Therefore, to prevent chronic MR occupancy by CORT, co-expression of HSD11β enzymes is essential. HSD11β2 converts CORT to its inactive metabolite, cortisone (CTC). HSD11β1 can catalyze this reaction in both directions; however, in the skin, HSD11β1 predominantly acts as a reductase, regenerating active CORT. In contrast, HSD11β2 catalyzes only CORT inactivation and is mainly expressed in mineralocorticoid-selective tissues such as the kidney and liver. Low HSD11β2 mRNA expression has also been reported in aortic endothelial cells [40].

In our study, HSD11β2 expression was slightly reduced in skin vessels of STZ rats. Reduced enzyme expression suggests increased MR activation by glucocorticoids. Thus, the pronounced decrease in HSD11β2 in STZ rats may reduce MR selectivity for ALDO, exposing the receptor to glucocorticoids and potentially enhancing vascular permeability under hyperglycemic conditions. This hypothesis is supported by data showing that cortisol increases paracellular permeability and alters ZO-1 distribution in HUVECs under normoglycemic conditions [41]. Moreover, in patients with type 2 diabetes, HSD11β2 activity is shifted toward higher intracellular cortisol exposure [42]. Importantly, the decrease in HSD11β2 expression in diabetic skin vessels suggests diminished MR selectivity for ALDO and increased receptor activation by glucocorticoids. This mechanism may contribute to long-term peripheral diabetic complications. Consequently, combining MR antagonists with topical glucocorticoids could offer therapeutic benefit in dermatopathies associated with diabetes.

We also found reduced HSD11β2 expression in the epidermis, hair follicles, and sebaceous glands of STZ rats. In contrast, other authors reported a severalfold increase in MR expression in intact skin and over wounds in STZ mice [36]. These differences suggest that HSD11β2 expression changes in diabetes are not confined to specific skin structures, and may depend on glycemic control and local or systemic ALDO concentrations. Co-expression of MR and HSD11β2 has been demonstrated in the epidermis and sweat glands of healthy human skin [31]. Therefore, reduced HSD11β2 activity may play an important role in the development of diabetic skin pathology.

Chronic EPL treatment did not alter mRNA expression or IHC staining for MR or HSD11β2 in the skin of STZ rats. There are, however, studies showing that subcutaneous spironolactone (a nonspecific MR antagonist) partially reversed the diabetes-induced reduction in renal 11β-HSD2 activity and gene expression in diabetic rats [43].

This study is the first to demonstrate, in a diabetic rat model, the distribution and hyperglycemia-induced changes in the expression of MR, HSD11β2, VEGF, vWF, and ZO-1 in specific skin structures, including blood vessels, the epidermis, hair follicles, and sebaceous glands. Previous work has shown MR expression in epidermal keratinocytes, hair follicles, and sebaceous and sweat glands in both human and mouse skin [31,44].

The well-established Miles assay was used to assess vascular permeability [21]. The method is based on the principle that factors increasing vascular permeability enhance the extravasation of Evans blue dye into the tissue. We observed a marked increase (approximately 77%) in the permeability of skin blood vessels in STZ-induced diabetic rats. Permeability was measured five weeks after diabetes induction; however, this phenomenon has been reported as early as one week after STZ injection and may persist for up to seven weeks [45,46]. Increased venular permeability has also been documented in the coronary microcirculation of STZ rats four weeks after the onset of diabetes [47]. Elevated albumin leakage, measured as ^125^I-albumin accumulation, has been shown in the eye, sciatic nerve, aorta, and kidney of biobreeding rats (a model of type 2 diabetes) and STZ rats—tissues prone to diabetic vascular injury in humans [48]. Elevated vascular permeability has been reported in the skin of STZ-treated rats [45,49], hyperglycemic and hyperinsulinemic UCP1/DTA transgenic mice [50], and in patients with type 1 diabetes [51,52,53]. A meta-analysis involving 470 individuals with type 1 and type 2 diabetes also confirmed reduced dermal microvascular function [54]. Our findings extend these observations to the skin microvasculature.

Our results indicate that the increase in skin vascular permeability in diabetes occurs through an MR-dependent mechanism. EPL significantly reduced skin vascular permeability in STZ rats. The efficacy of MR blockade in reducing vascular permeability has previously been demonstrated in the renal microcirculation of patients with type 1 and type 2 diabetes [55,56,57,58], as well as in STZ rats and db/db mice [39]. A meta-analysis of patients with various conditions, including type 2 diabetes, showed a positive association between increased skin vascular permeability and microalbuminuria, suggesting that similar mechanisms regulate permeability in the skin and kidney microcirculation [59].

Several pleiotropic actions of EPL may contribute to the reduction in skin vascular permeability observed in STZ rats. EPL decreases the production of inflammatory cytokines [23,60], and these effects appear to extend beyond classic MR antagonism [61]. In patients with type 2 diabetes receiving EPL (50–100 mg/day) together with enalapril, a significant reduction in albuminuria was reported, attributed to suppression of ALDO-induced inflammatory signaling [55]. In Dahl salt-sensitive rats, EPL also inhibited coronary vascular inflammation even at non-antihypertensive doses [62].

A second major mechanism involves the reduction of oxidative stress and the restoration of endothelial function [63]. In STZ rats and mice, both NO-dependent and EDH-type relaxations are diminished in small mesenteric arteries, accompanied by elevated oxidative stress [64,65]. While NO and PGI_2_ are dominant vasorelaxant mediators in large arteries, EDH, together with NO, plays a key role in resistance vessels, including small mesenteric arteries [66]. Given the functional similarity of resistance vessels across vascular beds, EDH-related mechanisms may also contribute to the protective effects of EPL in skin vessels. In diabetic mice, EPL reduced ROS generation by increasing the expression of antioxidant enzymes and enhancing soluble guanylyl cyclase β-subunit expression in the aorta [67]. EPL also improves L-arginine transport, and therefore NO synthesis, by modulating cationic amino acid transporter-1 in endothelial cells [68]. In a rat model of type 2 diabetes, EPL reduced mitochondrial ROS production without changing mitochondrial transcription factor A or nuclear respiratory factor-1 expression, suggesting that EPL-mediated ROS reduction involves non-genomic mechanisms, including MAP kinases, protein kinase C, and phospholipase C [69].

**Figure 12:**
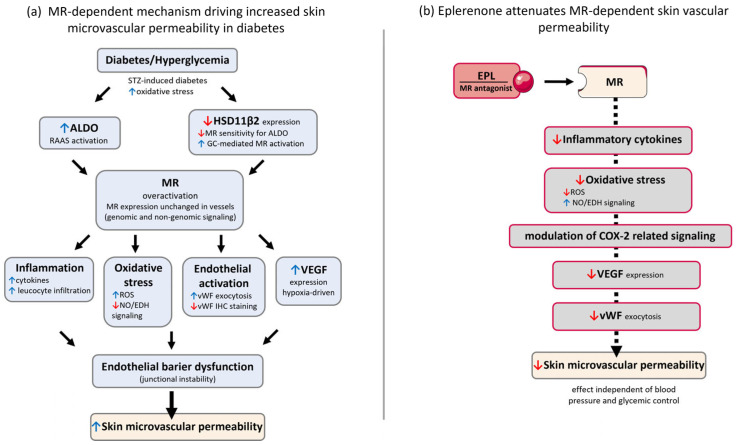
Mineralocorticoid receptor–dependent mechanisms of diabetic skin vascular permeability and their modulation by eplerenone. (**a**) Experimental diabetes and hyperglycemia are associated with increased aldosterone (ALDO) levels and reduced expression of 11β-hydroxysteroid dehydrogenase type 2 (HSD11β2) in the skin, leading to loss of mineralocorticoid receptor (MR) selectivity and enhanced MR activation by both mineralocorticoids and glucocorticoids. MR overactivation triggers genomic and non-genomic signaling pathways that promote inflammation, oxidative stress, vascular endothelial growth factor (VEGF) up-regulation, and endothelial activation characterized by increased von Willebrand factor (vWF) exocytosis. These processes converge to destabilize endothelial junctions, resulting in endothelial barrier dysfunction and increased skin microvascular permeability, which contributes to the development of diabetic skin disorders. (**b**) Pharmacological MR blockade with eplerenone (EPL) attenuates diabetes-induced skin vascular permeability. EPL reduces MR-dependent inflammatory signaling, oxidative stress, VEGF expression, and vWF exocytosis, thereby improving endothelial barrier function and limiting vascular leakage. These protective effects occur independently of changes in blood pressure or glycemic control, highlighting the pleiotropic vasculoprotective actions of EPL in diabetic skin microvasculature.

Further evidence for pleiotropic mechanisms comes from our previous work demonstrating that indomethacin attenuates the increase in ALDO-induced skin vascular permeability in normoglycemic rats [16]. Chronic ALDO administration has been shown to impair aortic endothelial function in both normotensive and hypertensive rats by activating COX-2 and promoting a vascular inflammatory phenotype in the myocardium and vascular smooth muscle cells [70,71,72]. In models of Ang II–induced hypertension, EPL reduced COX-2 expression in coronary vessels without changes in blood pressure or diuresis, indicating that ALDO regulates COX-2 expression independently of systemic hemodynamic effects [71]. Taken together, these findings indicate that EPL reduces vascular permeability in diabetic skin through a combination of mechanisms: MR inhibition, suppression of inflammatory pathways, reduction of oxidative stress, enhancement of NO- and EDH-dependent endothelial function, and modulation of COX-2–related signaling. Figure 12 illustrates the potential complex mineralocorticoid receptor–dependent mechanisms underlying diabetic skin vascular permeability and their modulation by eplerenone.

It is important to emphasize that microvessel permeability is strongly influenced by hemodynamic factors [73]. Vascular dysfunction is therefore regarded as a key contributor to impaired blood flow regulation in diabetes [74,75].

We did not observe any changes in blood pressure or heart rate following EPL treatment in STZ rats, although skin microcirculatory blood flow was not evaluated. In patients with type 1 diabetes, increased skin capillary pressure has been reported compared with age- and sex-matched normoglycemic controls [76]. Similarly, in SHR rats, even small increases in capillary pressure were shown to elevate net filtration and the escape rate of serum albumin [77]. In our study, the effect of EPL in reducing vascular permeability in STZ rats appears to be independent of its hypotensive or hypoglycemic actions, as no changes in blood pressure, heart rate, or glucose concentration were detected after 10 days of treatment. This observation is consistent with our previous findings showing that a 10-day EPL treatment at a dose of 100 mg/kg reduced arterial thrombosis in STZ-induced diabetic rats without affecting hemodynamic parameters [15,23], as well as with reports demonstrating that this dose is non-antihypertensive yet effective in attenuating vascular inflammation and endothelial dysfunction in other experimental models [62,78].

Next, we examined several factors that could contribute to MR/ALDO-dependent increases in skin vascular permeability. VEGF, a well-established mediator of vascular leakage, increases microvascular endothelial permeability through NO and prostacyclin, as both NOS and COX inhibitors attenuate its effects [10]. We previously demonstrated a VEGF-dependent rise in skin vascular permeability after acute ALDO administration in normoglycemic rats [16]. In the present study, VEGF expression in skin vessels was higher in STZ rats than in NORM animals. This increase is likely associated with diabetic microangiopathy and microembolization [74], which together promote tissue hypoxia [79,80]. Hypoxia is a strong stimulus for VEGF upregulation [79] and may therefore link diabetes-induced vascular injury with increased permeability. Elevated VEGF expression has also been reported in the retina, glomerular vasculature, and renal tubules in STZ-induced diabetes [81,82,83]. EPL treatment reduced VEGF staining intensity in both STZ and NORM rats, suggesting that decreased VEGF expression may contribute to the EPL-induced reduction in vascular permeability.

vWF has recently been identified as an inflammatory mediator capable of increasing vascular permeability through disruption of endothelial tight junctions [18]. vWF is stored in Weibel–Palade bodies (WPBs) and released upon endothelial activation [84]. In our study, diabetes reduced vWF IHC staining intensity in skin vessels. Although vWF protein staining was decreased, no differences in vWF mRNA expression were detected in skin homogenates from STZ and NORM rats, which may reflect the low proportion of endothelial cells in whole-tissue samples. Increased exocytosis of vWF from WPBs could explain the reduced intracellular staining observed in diabetic rats. A similar effect was reported in human aortic endothelial cells, in which spironolactone inhibited ALDO-induced vWF exocytosis [85]. Reports regarding vascular vWF staining in diabetes are inconsistent: some studies found no change in skin vessels of patients with type 1 diabetes [86], and no differences in retinal vessels from patients with diabetic retinopathy [87]. In our study, EPL reduced vWF exocytosis in both STZ and NORM rats, highlighting a link between elevated ALDO levels and vWF release in diabetes.

ALDO-induced increases in paracellular permeability have been associated with tight junction remodeling, including the formation of F-actin stress fibers and disruption of ZO-1 junctional strands in HUVECs under normoglycemic conditions [41]. In our study, ZO-1 expression in skin vessels did not differ between STZ and NORM rats, and EPL did not alter ZO-1 mRNA levels or IHC staining. Although we cannot exclude its involvement, microstructural ZO-1 distribution within endothelial cells was not assessed. In contrast to our findings, Kirsch et al. [41] reported increased ZO-1 expression after MR antagonism in HUVECs under normoglycemia, suggesting improved endothelial integrity. Endothelial junction proteins play a key role in maintaining vascular integrity [88]. ZO-1 anchors tight junction proteins to the cytoskeleton and stabilizes adherens junctions [19].

To further characterize ALDO-dependent regulation of permeability, we examined its effects on human dermal microvascular endothelial cells (HDMECs) under normoglycemic and hyperglycemic conditions. ALDO (10^−9^ M) increased endothelial permeability at both 15 and 60 min, with a stronger response under hyperglycemia, suggesting that elevated glucose enhances endothelial susceptibility to ALDO-induced barrier dysfunction. EPL (10^−5^ M) abolished the ALDO effect. Similar observations have been reported in HUVECs, in which ALDO increased permeability to 70 kDa dextran within 60 min and altered endothelial integrity through MR-dependent cytoskeletal and junctional rearrangements, as well as changes in eNOS activity [41]. Although barrier tightness varies among peripheral vascular beds [89], the effect of ALDO on endothelial permeability appears to be consistent and is particularly pronounced under hyperglycemic conditions. Our results suggest that pre-existing endothelial dysfunction may amplify cellular responses to ALDO. This study extends earlier findings by demonstrating both rapid (15 min; non-genomic) and delayed (60 min; genomic) ALDO effects on endothelial permeability under normal and high-glucose conditions. The lack of significant differences between normoglycemic and hyperglycemic control groups may be attributable to the in vitro nature of the experiments, where systemic, hormonal, and inflammatory factors contribute to hyperglycemia-associated alterations.

Vascular permeability in diabetes is a multifactorial process regulated by metabolic, inflammatory, and hormonal pathways, including mechanisms independent of ALDO/MR signaling. In the present study, the consistent reduction of vascular permeability by the selective MR antagonist eplerenone, observed both in vivo and in vitro, provides functional evidence supporting a role for ALDO/MR signaling in diabetic skin microcirculation. The use of complementary experimental models allowed partial control of non-ALDO-dependent factors: in vitro experiments minimized systemic confounders, whereas the in vivo model preserved the complex diabetic milieu. The absence of changes in systemic hemodynamic parameters or glycemia following eplerenone treatment suggests that the observed effects were not secondary to hypotensive or hypoglycemic actions. Together with previous reports demonstrating ALDO-induced increases in vascular permeability that are prevented by MR blockade, these findings support a meaningful contribution of ALDO/MR signaling to diabetic microvascular dysfunction.

## 5. Limitations

There are some limitations to our study. We used immunohistochemistry (IHC) to assess the expression of commonly evaluated markers related to vascular permeability. IHC is a well-established and widely accepted semi-quantitative technique in both clinical and experimental research; however, despite ongoing efforts toward standardization, no universally accepted method for fully quantitative post-analytical scoring exists [90]. Therefore, protein expression was assessed using a widely accepted semi-quantitative scoring system (−/+/++/+++) in a blinded evaluation by an experienced histologist. Although full numerical quantification and statistical comparison would further strengthen the findings, such re-analysis was not feasible due to the retrospective nature of the IHC experiments. For this reason, the IHC findings in the present study are interpreted as supportive and descriptive and are discussed in the context of complementary functional, biochemical, and morphological data rather than as standalone quantitative evidence. Consequently, IHC results should be considered supportive rather than definitive, and future studies using complementary quantitative methods, such as immunoblotting or real-time PCR, are warranted.

Another limitation concerns the sexually dimorphic activity of endothelial MR [91]. In female obese mice, aldosterone-mediated MR overactivation increases Na^+^ influx through ENaC channels and suppresses ERα-dependent eNOS activation, resulting in endothelial dysfunction. In males, MR overactivation primarily enhances the expression of adhesion molecules such as ICAM-1 and P-selectin, thereby promoting leukocyte adhesion and inflammation [92]. Although MR overactivation impairs eNOS in both sexes, the effect is less pronounced in males [91]. MR inhibition with MRAs may therefore promote ERα-dependent NO production, and some beneficial effects of EPL observed in females but not in males could be linked to differences in NO bioactivity [93,94].

Vascular permeability in diabetes is influenced by multiple mechanisms, including pathways independent of ALDO/MR signaling. Although our findings support the involvement of the ALDO/MR pathway, we cannot exclude the contribution of additional diabetes-related factors. Moreover, while adrenalectomized animal models could provide further mechanistic insight, their interpretability is limited by the concomitant elimination of other adrenal hormones and the persistence of local, tissue-specific ALDO/MR systems.

## 6. Conclusions

Using an in vivo model of insulin-dependent diabetes in rats (STZ rats), we demonstrated: (1) a significant increase in skin vascular permeability that was attenuated by the MR antagonist eplerenone (EPL); (2) reduced MR and HSD11β2 expression in the skin of STZ rats; (3) increased VEGF expression and enhanced vWF exocytosis from endothelial cells of skin vessels, both of which were diminished by EPL; (4) a markedly stronger ALDO-induced increase in endothelial permeability in human dermal microvascular endothelial cells under hyperglycemic conditions, which was prevented by EPL; and (5) higher expression of MR, HSD11β2, VEGF, vWF, and ZO-1 in several skin structures, including vessels, epidermis, hair follicles, and sebaceous glands, of STZ rats compared with normoglycemic controls. These results extend current knowledge of the pathological effects of ALDO on the vasculature by demonstrating its role in regulating the skin’s vascular permeability under hyperglycemic conditions.

## Figures and Tables

**Table 1:** Effect of diabetes and/or eplerenone administration on the percentage ratio of individual skin layers.

Group		Percentage of Total Skin Thickness [%]		
		Epidermis	Dermis	Subcataneous Tissue
NORM	CON	2.03 ± 0.98 a	88.77 ± 10.8 a	9.20 ± 2.72 a
	EPL	2.65 ± 0.76 a	88.75 ± 9.36 a	8.60 ± 1.15 a
STZ	CON	5.22 ± 2.64 b	81.04 ± 5.24 b	13.74 ± 2.35 b
	EPL	6.99 ± 4.21 b	76.63 ± 9.31 b	16.38 ± 3.11 c

CON—eplerenone solvent; EPL—eplerenone; NORM—normoglycemic group; STZ—diabetic group. Results are presented as mean ± SD; n = 6–9. Statistical relationships among groups were visualized using compact letter displays, where groups sharing the same letter are not significantly different, whereas groups without a common letter differ significantly (*p* < 0.05).

**Table 2:** Relative expression of the studied genes normalized to β-actin.

Group		MR/Actb [%]	HSD11β2/Actb [%]	vWF/Actb [%]	VEGF/Actb [%]	ZO-1/Actb [%]
NORM	CON	18.64 ± 0.97 a	18.48 ± 1.19 ab	20.57 ± 0.32 a	19.27 ± 1.90 a	19.70 ± 2.71 a
	EPL	18.49 ± 2.61 a	18.35 ± 2.26 a	20.67 ± 0.62 a	19.29 ± 3.13 a	18.35 ± 0.76 a
STZ	CON	9.35 ± 1.76 b	13.36 ± 1.74 bc	20.07 ± 0.36 a	17.32 ± 1.77 a	18.18 ± 2.00 a
	EPL	8.08 ± 1.01 c	11.77 ± 3.32 c	20.09 ± 0.58 a	16.43 ± 0.83 a	17.30 ± 1.50 a

Actb—β-actin; CON—eplerenone solvent; EPL—eplerenone; HSD11β2—11β hydroxysteroid dehydrogenase; MR—mineralocorticoid receptor; NORM—normoglycemic group; STZ—diabetic group; VEGF—vascular endothelial growth factor; vWF—von Willebrand factor; ZO-1—zonula occludens 1. Results are presented as mean ± SD; n = 7–8. Statistical relationships among groups were visualized using compact letter displays, where groups sharing the same letter are not significantly different, whereas groups without a common letter differ significantly (*p* < 0.05).

## Data Availability

The raw data supporting the findings of this manuscript will be provided by the authors at any time to the reviewers, and thereafter, to any researcher after publication.

## Supplementary Materials

The following supporting information can be downloaded at: https://www.mdpi.com/article/10.3390/cells15010089/s1, Figure S1. Effect of diabetes on the microscopic image of skin cross-sections with H+E staining (×100); Figure S2. Effect of diabetes on the microscopic image of skin with immunohistochemical staining for the mineralocorticoid receptor (MR); Figure S3. Effect of diabetes on the microscopic image of skin with immunohistochemical staining for 11β-hydroxysteroid dehydrogenase (HSD11β2); Figure S4. Effect of diabetes on the microscopic image of skin with immunohistochemical staining for vascular endothelial growth factor (VEGF); Figure S5. Effect of diabetes on the microscopic image of skin with immunohistochemical staining for von Willebrand factor (vWF); Figure S6. Effect of diabetes on the microscopic image of skin stained immunohistochemically for zonula occludens-1 (ZO-1); Figure S7. Permeability of the human dermal microvascular endothelial cell (HMEC-1) monolayer under normal (NORM) or hyperglycemic (HG) conditions; Table S1. Effect of diabetes and/or eplerenone administration on general physiological parameters; Table S2. Effect of diabetes and/or eplerenone administration on basic morphological parameters.

## Abbreviations

The following abbreviations are used in this manuscript:

ALDO	Aldosterone
BW	Body weight
CON	Vehicle
DBP	Diastolic blood pressure
DERM	Dermis
EB	Evans blue
EPI	Epidermis
EPL	Eplerenone
GLU	Glucose
GR	Glucocorticoid receptor
HCT	Hematocrit
HF	Hair follicle
HG	Hyperglycemic
HGB	Hemoglobin
HMEC-1	Human dermal microvascular endothelial cells
HR	Heart rate
HSD11β2	11β-hydroxysteroid dehydrogenase type 2
HUVEC	Human umbilical vein endothelial cell
IHC	Immunohistochemistry
I.V.	Intravenous
MCH	Mean mass of hemoglobin in a red cell
MCHC	Mean concentration of hemoglobin in a red cell
MCV	Red cell volume
MR	Mineralocorticoid receptor
MSC	Panniculus carnosus muscle
NO	Nitric oxide
NORM	Normoglycemic, control animals
PLT	Platelets
P.O.	Per os
RAAS	Renin–angiotensin–aldosterone system
RBC	Red blood cells
ROS	Reactive oxygen species
rt-qPCR	Real-time quantitative polymerase chain reaction
SBP	Systolic blood pressure
SEB	Sebaceous gland
STZ	Streptozotocin
SC	Subcutaneous tissue
VEGF	Vascular endothelial growth factor
vWF	von Willebrand factor
WBC	White blood cells
ZO-1	Tight junction protein

## References

[1] Aleksiejczuk M., Gromotowicz-Poplawska A., Marcinczyk N., Przylipiak A., Chabielska E. (2019). The expression of the renin-angiotensin-aldosterone system in the skin and its effects on skin physiology and pathophysiology. J. Physiol. Pharmacol..

[2] Pérez P. (2022). The mineralocorticoid receptor in skin disease. Br. J. Pharmacol..

[3] Fossas De Mello N., Bollag W.B. (2025). The role of the mineralocorticoid receptor in skin. Mol. Cell Endocrinol..

[4] Ruhs S., Nolze A., Hübschmann R., Grossmann C. (2017). 30 Years of the Mineralocorticoid Receptor: Nongenomic effects via the mineralocorticoid receptor. J. Endocrinol..

[5] Ibarrola J., Jaffe I.Z. (2024). The Mineralocorticoid Receptor in the Vasculature: Friend or Foe?. Annu. Rev. Physiol..

[6] Ferreira N.S., Tostes R.C., Paradis P., Schiffrin E.L. (2021). Aldosterone, Inflammation, Immune System, and Hypertension. Am. J. Hypertens..

[7] Boix J., Sevilla L.M., Sáez Z., Carceller E., Pérez P. (2016). Epidermal Mineralocorticoid Receptor Plays Beneficial and Adverse Effects in Skin and Mediates Glucocorticoid Responses. J. Investig. Dermatol..

[8] Concistrè A., Petramala L., Bonvicini M., Gigante A., Collalti G., Pellicano C., Olmati F., Iannucci G., Soldini M., Rosato E. (2020). Comparisons of skin microvascular changes in patients with primary aldosteronism and essential hypertension. Hypertens. Res..

[9] Wautier J.L., Wautier M.P. (2022). Vascular permeability in diseases. Int. J. Mol. Sci..

[10] Murohara T., Horowitz J.R., Silver M., Tsurumi Y., Chen D., Sullivan A., Isner J.M. (1998). Vascular endothelial growth factor/vascular permeability factor enhances vascular permeability via nitric oxide and prostacyclin. Circulation.

[11] Canonica J., Mehanna C., Bonnard B., Jonet L., Gelize E., Jais J.P., Jaisser F., Zhao M., Behar-Cohen F. (2019). Effect of acute and chronic aldosterone exposure on the retinal pigment epithelium-choroid complex in rodents. Exp. Eye Res..

[12] Hollenberg N.K., Stevanovic R., Agarwal A., Lansang M.C., Price D.A., Laffel L.M., Williams G.H., Fisher N.D. (2004). Plasma aldosterone concentration in the patient with diabetes mellitus. Kidney Int..

[13] Gromotowicz-Poplawska A., Szoka P., Zakrzeska A., Kolodziejczyk P., Marcinczyk N., Szemraj J., Tutka P., Chabielska E. (2021). Hyperglycemia Potentiates Prothrombotic Effect of Aldosterone in a Rat Arterial Thrombosis Model. Cells.

[14] Mundi S., Massaro M., Scoditti E., Carluccio M.A., van Hinsbergh V.M., Iruela-Arispe M.L., De Caterina R. (2018). Endothelial permeability, LDL deposition, and cardiovascular risk factors-a review. Cardiovasc. Res..

[15] Gromotowicz-Poplawska A., Kloza M., Aleksiejczuk M., Marcinczyk N., Szemraj J., Kozlowska H., Chabielska E. (2019). Nitric oxide as a modulator in platelet- and endothelium-dependent antithrombotic effect of eplerenone in diabetic rats. J. Physiol. Pharmacol..

[16] Aleksiejczuk M., Gromotowicz-Poplawska A., Marcinczyk N., Stelmaszewska J., Dzieciol J., Chabielska E. (2022). Aldosterone increases vascular permeability in rat skin. Cells.

[17] Odermatt A., Kratschmar D.V. (2012). Tissue-specific modulation of mineralocorticoid receptor function by 11β-hydroxysteroid dehydrogenases: An overview. Mol. Cell Endocrinol..

[18] Gragnano F., Sperlongano S., Golia E., Natale F., Bianchi R., Crisci M., Fimiani F., Pariggiano I., Diana V., Carbone A. (2017). The Role of von Willebrand Factor in Vascular Inflammation: From Pathogenesis to Targeted Therapy. Mediat. Inflamm..

[19] Tornavaca O., Chia M., Dufton N., Almagro L.O., Conway D.E., Randi A.M., Schwartz M.A., Matter K., Balda M.S. (2015). ZO-1 controls endothelial adherens junctions, cell-cell tension, angiogenesis, and barrier formation. J. Cell Biol..

[20] Jones-Bolin S. (2012). Guidelines for the care and use of laboratory animals in biomedical research. Curr. Protoc. Pharmacol..

[21] Brash J.T., Ruhrberg C., Fantin A. (2018). Evaluating vascular hyperpermeability-inducing agents in the skin with the miles assay. J. Vis. Exp..

[22] Hilfenhaus M. (1976). Circadian rhythm of the renin-angiotensin-aldosterone system in the rat. Arch. Toxicol..

[23] Zakrzeska A., Gromotowicz-Popławska A., Szemraj J., Szoka P., Kisiel W., Purta T., Kasacka I., Chabielska E. (2015). Eplerenone reduces arterial thrombosis in diabetic rats. J. Renin Angiotensin Aldosterone Syst..

[24] Miles A.A., Miles E.M. (1952). Vascular reactions to histamine, histamine-liberator and leukotoxin in the skin of guinea-pigs. J. Physiol..

[25] Hellemans J., Mortier G., De Paepe A., Speleman F., Vandesompele J. (2007). qBase relative quantification framework and software for management and automated analysis of real-time quantitative PCR data. Genome Biol..

[26] Reynoso-Palomar A., Mena-Aguilar G., Cruz-García M., Pastelín-Rojas C., Villa-Mancera A. (2017). Production of aldosterone in cardiac tissues of healthy dogs and with dilated myocardiopathy. Vet. World.

[27] Chen X.F., Lin W.D., Lu S.L., Xie T., Ge K., Shi Y.Q., Zou J.J., Liu Z.M., Liao W.Q. (2010). Mechanistic study of endogenous skin lesions in diabetic rats. Exp. Dermatol..

[28] Andrade T.A.M., Masson-Meyers D.S., Caetano G.F., Terra V.A., Ovidio P.P., Jordão-Júnior A.A., Frade M.A.C. (2017). Skin changes in streptozotocin-induced diabetic rats. Biochem. Biophys. Res. Commun..

[29] Hao S.Y., Ren M., Yang C., Lin D.Z., Chen L.H., Zhu P., Cheng H., Yan L. (2011). Activation of skin renin-angiotensin system in diabetic rats. Endocrine.

[30] Cook C.S., Zhang L., Ames G.B., Fischer J., Zhang J., Levin S. (2003). Single- and repeated-dose pharmacokinetics of eplerenone, a selective aldosterone receptor blocker, in rats. Xenobiotica.

[31] Kenouch S., Lombes M., Delahaye F., Eugene E., Bonvalet J.P., Farman N. (1994). Human skin as target for aldosterone: Coexpression of mineralocorticoid receptors and 11 beta-hydroxysteroid dehydrogenase. J. Clin. Endocrinol. Metab..

[32] Steckelings U.M., Czarnelzki B.M. (1995). The renin-angiotensin-system in the skin. Evidence for its presence and possible functional implications. Exp. Dermatol..

[33] Takeda Y. (2004). Vascular synthesis of aldosterone: Role in hypertension. Mol. Cell Endocrinol..

[34] Ackermann D., Jamin H., Klossner R. (2019). Mineralocorticoid receptor and diabetes: In favor of other corticosteroids than aldosterone. Endocr. Abstr..

[35] Tafelski S., Doaa M., Mohammed S. (2019). Identification of mineralocorticoid and glucocorticoid receptors on peripheral nociceptors: Translation of experimental findings from animal to human biology. Brain Res..

[36] Nguyen V.T., Farman N., Maubec E., Nassar D., Desposito D., Waeckel L., Aractingi S., Jaisser F. (2016). Re-Epithelialization of Pathological Cutaneous WoundsIs Improved by Local Mineralocorticoid Receptor Antagonism. J. Investig. Dermatol..

[37] Zhao M., Gelize E., Levy R., Moulin A., Azan F., Berdugo M., Naud M.C., Guegan J., Delaunay K., Pussard E. (2021). Mineralocorticoid Receptor Pathway and Its Antagonism in a Model of Diabetic Retinopathy. Diabetes.

[38] Kosugi T., Heinig M., Nakayama T., Matsuo S., Nakagawa T. (2010). eNOS knockout mice with advanced diabetic nephropathyhave less benefit from renin-angiotensin blockade than from aldosterone receptor antagonists. Am. J. Pathol..

[39] Guo C., Martinez-Vasquez D., Mendez G.P., Toniolo M.F., Yao T.M., Oestreicher E.M., Kikuchi T., Lapointe N., Pojoga L., Williams G.H. (2006). Mineralocorticoid receptor antagonist reduces renal injury in rodent models of types 1 and 2 diabetes mellitus. Endocrinology.

[40] Baker M.E., Yoshinao K. (2017). 30 Years of the Mineralocorticoid Receptor: Evolution of the mineralocorticoid receptor: Sequence, structure and function. J. Endocrinol..

[41] Kirsch T., Beese M., Wyss K., Klinge U., Haller H., Haubitz M., Fiebeler A. (2013). Aldosterone modulates endothelial permeability and endothelial nitric oxide synthase activity by rearrangement of the actin cytoskeleton. Hypertension.

[42] Gant C.M., Minovic I., Binnenmars H., de Vries L., Kema I., van Beek A., Navis G., Bakker S., Laverman G.D. (2018). Lower Renal Function Is Associated With Derangement of 11- β Hydroxysteroid Dehydrogenase in Type 2 Diabetes. J. Endocr. Soc..

[43] Liu Y., Rajur K., Tolbert E., Dworkin L.D. (2000). Endogenous hepatocyte growth factor ameliorates chronic renal injury by activating matrix degradation pathways. Kidney Int..

[44] Sainte Marie Y., Toulon A., Paus R., Maubec E., Cherfa A., Grossin M., Descamps V., Clemessy M., Gasc J.M., Peuchmaur M. (2007). Targeted Skin Overexpression of the Mineralocorticoid Receptor in Mice Causes Epidermal Atrophy, Premature Skin Barrier Formation, Eye Abnormalities, and Alopecia. Am. J. Pathol..

[45] Lawson S.R., Gabra B.H., Guérin B., Neugebauer W., Nantel F., Battistini B., Sirois P. (2005). Enhanced dermal and retinal vascular permeability in streptozotocin-induced type 1 diabetes in Wistar rats: Blockade with a selective bradykinin B1receptor antagonist. Regul. Pept..

[46] Chakir M., Plante G.E., Maheux P. (1998). Reduction of capillary permeability in the fructose-induced hypertensive rat. Am. J. Hypertens..

[47] Yuan S.Y., Ustinova E.E., Wu M.H., Tinsley J.H., Xu W., Korompai F.L., Taulman A.C. (2000). Protein kinase C activation contributes to microvascular barrier dysfunction in the heart at early stages of diabetes. Circ. Res..

[48] Williamson J.R., Chang K., Tilton R.G., Prater C., Jeffrey J.R., Weigel C., Sherman W.R., Eades D.M., Kilo C. (1987). Increased vascular permeability in spontaneously diabetic BB/W rats and in rats with mild versus severe streptozocin-induced diabetes. Prevention by aldose reductase inhibitors and castration. Diabetes.

[49] Chakir M., Plante G.E.E. (1996). Endothelial dysfunction in diabetes mellitus. Prostaglandins Leukot. Essent. Fat. Acids.

[50] Algenstaedt P., Schaefer C., Biermann T., Hamann A., Schwarzloh B., Greten H., Rüther W., Hansen-Algenstaedt N. (2003). Microvascular alterations in diabetic mice correlate with level of hyperglycemia. Diabetes.

[51] Oomen P.H., Jager J., Hoogenberg K., Dullaart R.P., Reitsma W.D., Smit A.J. (1999). Capillary permeability is increased in normo- and microalbuminuric Type 1 diabetic patients: Amelioration by ACE-inhibition. Eur. J. Clin. Investig..

[52] Lefrandt J.D., Bosma E., Oomen P.H.N., Hoeven J.H., Roon A.M., Smit A.J., Hoogenberg K. (2003). Sympathetic mediated vasomotion and skin capillary permeability in diabetic patients with peripheral neuropathy. Diabetologia.

[53] de Leeuw K., Kusumanto Y., Smit A.J., Oomen P., van der Hoeven J.H., Mulder N.H., Hospers G.A. (2008). Skin capillary permeability in the diabetic foot with critical limb ischaemia: The effects of a phVEGF165 gene product. Diabet. Med..

[54] Fuchs D., Dupon P.P., Schaap L.A., Draijer R. (2017). The association between diabetes and dermal microvascular dysfunction non-invasively assessed by laser Doppler with local thermal hyperemia: A systematic review with meta-analysis. Cardiovasc. Diabetol..

[55] Epstein M., Williams G.H., Weinberger M., Lewin A., Krause S., Mukherjee R., Patni R., Beckerman B. (2006). Selective aldosterone blockade with eplerenone reduces albuminuria in patients with type 2 diabetes. Clin. J. Am. Soc. Nephrol..

[56] Nielsen S.E., Persson F., Frandsen E., Sugaya T., Hess G., Zdunek D., Shjoedt K.J., Parving H.H., Rossing P. (2012). Spironolactone diminishes urinary albumin excretion in patients with type 1 diabetes and microalbuminuria: A randomized placebo-controlled crossover study. Diabet. Med..

[57] Schjoedt K.J., Rossing K., Juhl T.R., Boomsma F., Rossing P., Tarnow L., Parving H.H. (2005). Beneficial impact of spironolactone in diabetic nephropathy. Kidney Int..

[58] Rossing K., Schjoedt K.J., Smidt U.M., Boomsma F., Parving H.H. (2005). Beneficial effects of adding spironolactone torecommended antihypertensive treatment in diabetic nephropathy: A randomized, double-masked, cross-over study. Diabetes Care.

[59] Martens R.J.H., Henry R.M.A., Houben A.J.H.M., van der Kallen C.J., Kroon A.A., Schalkwijk C.G., Schram M.T., Sep S.J., Schaper N.C., Dagnelie P.C. (2016). Capillary rarefaction associates with albuminuria: The Maastricht study. J. Am. Soc. Nephrol..

[60] Ukimura A., Limori A. (2010). Pleiotropic Effects of Eplerenone on Murine Coxsackievirus B3 Myocarditis. J. Card. Fail..

[61] Pitt B., Remme W., Zannad F., Neaton J., Martinez F., Roniker B., Bittman R., Hurley S., Kleiman J., Gatlin M. (2003). Eplerenone, a Selective Aldosterone Blocker, in Patients with Left Ventricular Dysfunction after Myocardial Infarction. N. Engl. J. Med..

[62] Nagata K., Obata K., Xu J., Ichihara S., Noda A., Kimata H., Kato T., Izawa H., Murohara T., Yokota M. (2006). Mineralocorticoid receptor antagonism attenuates cardiac hypertrophy and failure in low-aldosterone hypertensive rats. Hypertension.

[63] Kratz M.T., Schirmer S.H., Baumhakel M., Bohm M. (2016). Improvement of endothelial function in a murine model of mild cholesterol-induced atherosclerosis by mineralocorticoid antagonism. Atherosclerosis.

[64] Leo C.H., Hart J.L., Woodman O.L. (2011). Impairment of both nitric oxide-mediated and EDHF-type relaxation in small mesenteric arteries from rats with streptozotocin-induced diabetes. Br. J. Pharmacol..

[65] Matsumoto T., Miyamori K., Kobayashi T., Kamata K. (2006). Specific impairment of endothelium-derived hyperpolarizing factor-type relaxation in mesenteric arteries from streptozotocin-induced diabetic mice. Vasc. Pharmacol..

[66] Kloza M., Baranowska-Kuczko M., Malinowska B., Karpińska O., Harasim-Symbor E., Kasacka I., Kozłowska H. (2017). The influence of DOCA-salt hypertension and chronic administration of the FAAH inhibitor URB597 on K(Ca)2.3/K(Ca)3.1-EDH-type relaxation in rat small mesenteric arteries. Vasc. Pharmacol..

[67] Silva M.A., Bruder-Nascimento T., Cau S.B., Lopes R.A., Mestriner F.L., Fais R.S., Touyz R.M., Tostes R.C. (2015). Spironolactone treatment attenuates vascular dysfunction in type 2 diabetic mice by decreasing oxidative stress and restoring NO/GC signaling. Front. Physiol..

[68] Shashar M., Hod T., Chernichovski T., Angel A., Kazan S., Grupper A., Naveh S., Kliuk-Ben Bassat O., Weinstein T., Schwartz I.F. (2018). Mineralocorticoid receptor blockade improves argininetransport and nitric oxide generation through modulation of cationic amino acid transporter-1 in endothelial cells. Nitric Oxide.

[69] Ramírez E., Klett-Mingo M., Ares-Carrasco S., Picatoste B., Ferrarini A., Rupérez F.J., Caro-Vadillo A., Barbas C., Egido J., Tuñón J. (2013). Eplerenone attenuated cardiac steatosis, apoptosis and diastolic dysfunction in experimental type-II diabetes. Cardiovasc. Diabetol..

[70] Blanco-Rivero J., Cachofeiro V., Lahera V., Aras-Lopez R., Márquez-Rodas I., Salaices M., Xavier F.E., Ferrer M., Balfagón G. (2005). Participation of prostacyclin in endothelial dysfunction induced by aldosterone in normotensive and hypertensive rats. Hypertension.

[71] Rocha R., Martin-Berger C.L., Yang P., Scherrer R., Delyani J., McMahon E. (2002). Selective Aldosterone Blockade Prevents Angiotensin II/Salt-Induced Vascular Inflammation in the Rat Heart. Endocrinology.

[72] Zhu C.J., Wang Q.Q., Zhou J.L., Liu H.Z., Hua F., Yang H.Z., Hu Z.W. (2012). The mineralocorticoid receptor-p38MAPK-NFκB or ERK-Sp1 signal pathways mediate aldosterone-stimulated inflammatory and profibrotic responses in rat vascular smooth muscle cells. Acta Pharmacol. Sin..

[73] Plante G.E., Chakir M., Ettaouil K., Lehoux S., Sirois P. (1996). Consequences of alteration in capillary permeability. Can. J. Physiol. Pharmacol..

[74] Parving H. (1991). Impact of Blood Pressure and Antihypertensive Treatment on Incipient and Overt Nephropathy, Retinopathy, and Endothelial Permeability in Diabetes Mellitus Available to Purchase. Diabetes Care.

[75] De Vriese A.S., Verbeuren T.J., Van De Voorde J., Lameire N.H., Vanhoutte P.M. (2000). Endothelial dysfunction in diabetes. Br. J. Pharmacol..

[76] Sandeman D.D., Shore A.C., Tooke J.E. (1992). Relation of skin capillary pressure in patients with insulin-dependent diabetes mellitus to complications and metabolic control. N. Engl. J. Med..

[77] Rippe B., Folkow B. (1977). Capillary permeability to albumin in normotensive and spontaneously hypertensive rats. Acta Physiol. Scand..

[78] Schafer A., Vogt C., Fraccarollo D., Widder J., Flierl U., Hildemann S.K., Ertl G., Bauersachs J. (2010). Eplerenone improves vascular function and reduces platelet activation in diabetic rats. J. Physiol. Pharmacol..

[79] Schoch H.J., Fischer S., Marti H.H. (2002). Hypoxia-induced vascular endothelial growth factor expression causes vascular leakage in the brain. Brain.

[80] Kim B.S., Chen J., Weinstein T., Noiri E., Goligorsky M.S. (2002). VEGF expression in hypoxia and hyperglycemia: Reciprocal effect on branching angiogenesis in epithelial-endothelial co-cultures. J. Am. Soc. Nephrol..

[81] Gilbert R.E., Vranes D., Berka J.L., Kelly D.J., Cox A., Wu L.L., Stacker S.A., Cooper M.E. (1998). Vascular endothelial growth factor and its receptors in control and diabetic rat eyes. Lab. Investig..

[82] Hammes H.P., Lin J., Bretzel R.G., Brownlee M., Breier G. (1998). Upregulation of the vascular endothelial growth factor/vascular endothelial growth factor receptor system in experimental background diabetic retinopathy of the rat. Diabetes.

[83] Cooper M.E., Vranes D., Youssef S., Stacker S.A., Cox A.J., Rizkalla B., Casley D.J., Bach L.A., Kelly D.J., Gilbert R.E. (1999). Increased renal expression of vascular endothelial growth factor (VEGF) and its receptor VEGFR-2 in experimental diabetes. Diabetes.

[84] McCormack J.J., da Silva M.L., Ferraro F., Patella F., Cutler D.F. (2017). Weibel-Palade bodies at a glance. J. Cell Sci..

[85] Jeong Y., Chaupin D.F., Matsushita K., Yamakuchi M., Cameron S.J., Morrell C.N., Lowenstein C.J. (2009). Aldosterone activates endothelial exocytosis. Proc. Natl. Acad. Sci. USA.

[86] Properzi G., Terenghi G., Gu X.H., Poccia G., Pasqua R., Francavilla S., Polak J.M. (1995). Early increase precedes a depletion of endothelin-1but not of von Willebrand factor in cutaneous microvessels of diabetic patients. A quantitative immunohistochemical study. J. Pathol..

[87] Boeri D., Cagliero E., Podestá F., Lorenzi M. (1994). Vascular wall von Willebrand factor in human diabetic retinopathy. Investig. Ophthalmol. Vis. Sci..

[88] Le Guelte A., Gavard J. (2011). Role of endothelial cell-cell junctions in endothelial permeability. Permeability Barrier: Methods and Protocols.

[89] Clyne A.M. (2021). Endothelial response to glucose: Dysfunction, metabolism, and transport. Biochem. Soc. Trans..

[90] Fedchenko N., Reifenrath J. (2014). Different approaches for interpretation and reporting of immunohistochemistry analysis results in the bone tissue—A review. Diagn. Pathol..

[91] Faulkner J.L., Kennard S., Huby A.C., Antonova G., Lu Q., Jaffe I.Z., Patel V.S., Fulton D.J.R., Belin de Chantemèle E.J. (2019). Progesterone predisposes females to obesity-associated leptin-mediated endothelial dysfunction via upregulating endothelial MR (Mineralocorticoid Receptor) expression. Hypertension.

[92] Wolter N.L., Jaffe I.Z. (2023). Emerging vascular cell-specific roles for mineralocorticoid receptor: Implications for understanding sex differences in cardiovascular disease. Am. J. Physiol. Physiol..

[93] Kane M.O., Anselm E., Rattmann Y.D., Auger C., Schini-Kerth V.B. (2009). Role of gender and estrogen receptors in the rat aorta endothelium-dependent relaxation to red wine polyphenols. Vasc. Pharmacol..

[94] Bielicka N., Misztal T., Chabielska E., Gromotowicz-Poplawska A. (2023). Sex-dependent effects of finerenone on hemostasis in normoglycemic and streptozotocin-induced diabetic mice. Biomed. Pharmacother..

