# Supplementary material for: The Role of Aldosterone in Vascular Permeability in Diabetes
Source: Cells. 2026 Jan 5;15(1):89. doi: 10.3390/cells15010089 (PMC12785615; doi:10.3390/cells15010089)
Supplement: Supplementary file 1 [file cells-15-00089-s001.zip › Suppl. Tables - revised.pdf]

Table S1. Effect of diabetes and/or eplerenone administration on general physiological parameters.

		NORM		STZ	
		**CON**	**EPL**	**CON**	**EPL**
Number of animals	n	16	16	20	20
BW (day 0)	[g]	302 ± 36	287 ± 52	314 ± 40	306 ± 22
BW (day 35)	[g]	361 ± 16	370 ± 32	**244 ± 40****	237 ± 31
BW (day 45)	[g]	371 ± 52	374 ± 76	**201 ± 40*****	214 ± 89
GLU (day 0)	[mg/dl]	68 ± 12	74 ± 36	72 ± 13	70 ± 36
GLU (day 45)	[mg/dl]	65 ± 60	77 ± 44	**285 ± 188*****	246 ± 125
SBP	mmHg	125 ± 8	119 ± 12	118 ± 9	120 ± 18
DBP	mmHg	106 ± 4	99 ± 8	102 ± 9	100 ± 18
HR	beats/min	410 ± 40	390 ± 80	380 ± 134	360 ± 89
Survival rate	[%]	100	100	80	90

BW – body weight; CON – eplerenone solvent; DBP – diastolic blood pressure; EPL – eplerenone; GLU – glucose; HR – heart rate; NORM – normoglycemic group; SBP – systolic blood pressure; STZ – diabetic group. n=16-20; **p<0.01; ***p<0.001 vs STZ+CON. Results are presented as mean ± SD.

Table S2. Effect of diabetes and/or eplerenone administration on basic morphological parameters.

	**WBC**	**RBC**	**HGB**	**HCT**	**MCV**	**MCH**	**MCHC**	**PLT**
	n ×10^3	n ×10^6	[g/dl]	[%]	[μm^3]	[pg]	[g/dl]	n ×10^6
**NORM + CON**	3.38 ± 1.10	8.79 ± 0.51	16.12 ± 0.86	46.08 ± 4.09	52.50 ± 2.35	18.03 ± 0.64	34.58 ± 0.78	655.0 ± 75.84
**NORM + EPL**	3.47 ± 0.48	8.45 ± 0.54	15.80 ± 0.40	46.13 ± 1.50	55.50 ± 3.20	19.69 ± 1.33	34.38 ± 0.51	644.8 ± 33.69
**STZ + CON**	**1.51 ± 0.57***	8.93 ± 1.32	16.44 ± 2.34	47.82 ± 7.05	53.80 ± 2.28	18.46 ± 0.69	34.05 ± 1.41	**416.6 ± 134.3****
**STZ + EPL**	1.91 ± 0.96	8.91 ± 0.91	16.34 ± 1.19	48.02 ± 2.74	54.33 ± 4.04	18.43 ± 1.16	34.02 ± 0.71	486.7± 171.6

CON – eplerenone solvent; EPL – eplerenone; HCT – hematocrit; HGB – hemoglobin; MCH – mean mass of hemoglobin in a red cell; MCHC – mean concentration of hemoglobin in a red cell; MCV – mean red cell volume; NORM – normoglycemic group; PLT – platelets; RBC – red blood cells; STZ – diabetic group; WBC – white blood cells. n=6-9; *p<0.05, **p<0.01, vs NORM+CON. Results are presented as mean ± SD.
