# Supplementary material for: The Role of Aldosterone in Vascular Permeability in Diabetes
Source: Cells. 2026 Jan 5;15(1):89. doi: 10.3390/cells15010089 (PMC12785615; doi:10.3390/cells15010089)
Supplement: Supplementary file 1 [file cells-15-00089-s001.zip › Legends for Supplemental Figures - revised.pdf]

LEGENDS FOR SUPPLEMENTAL FIGURES

Figure S1. Effect of diabetes on the microscopic image of skin cross-sections with H+E staining (×100). In the STZ group, marked dermal atrophy was observed, including areas with degenerating collagen structure. NORM – normoglycemic group; STZ – diabetic group; (epi) – epidermis; (hf) – hair follicle; (seb) – sebaceous gland; (msc) – panniculus carnosus muscle; (sc) – subcutaneous tissue. Magnification ×200.

Figure S2. Effect of diabetes on the microscopic image of skin with immunohistochemical staining for the mineralocorticoid receptor (MR). A positive antigen–antibody reaction appears as a tan-brown color. Staining was present in the epidermis, blood vessels, hair follicles, and sebaceous glands. In the STZ group, staining intensity was reduced (+) compared with the NORM group (++). CON – eplerenone solvent; EPL – eplerenone; NORM – normoglycemic group; STZ – diabetic group; (epi) – epidermis; (hf) – hair follicle; (seb) – sebaceous gland; (msc) – panniculus carnosus muscle; (fat) – adipose tissue; (ves) – blood vessel; (+) – poor staining; (++) – moderate staining. Magnification ×200.

Figure S3. Effect of diabetes on the microscopic image of skin with immunohistochemical staining for 11β-hydroxysteroid dehydrogenase (HSD11β2). A positive antigen–antibody reaction appears as a tan-brown color. Staining was present in the epidermis, blood vessels, hair follicles, and sebaceous glands. In the STZ group, staining intensity was reduced (+) compared with the NORM group (++). CON – eplerenone solvent; EPL – eplerenone; NORM – normoglycemic group; STZ – diabetic group; (epi) – epidermis; (hf) – hair follicle; (seb) – sebaceous gland; (msc) – panniculus carnosus muscle; (fat) – adipose tissue; (ves) – blood vessel; (+) – poor staining ; (++) – moderate staining. Magnification ×200.

Figure S4. Effect of diabetes on the microscopic image of skin with immunohistochemical staining for vascular endothelial growth factor (VEGF). A positive antigen-antibody reaction is seen as a tanbrown color. The staining in the NORM and STZ groups was observed only within the blood vessels, so the intensity of the staining in the skin section was assessed as lack of staining (-). NORM – normoglycemic group; STZ – diabetic group; (epi) – epidermis; (hf) – hair follicle; (sg) – sebaceous gland; (fat) – subcutaneous tissue; (msc) – panniculus carnosus; (ves) – blood vessel; (-) no color reaction. Magnification ×200.

Figure S5. Effect of diabetes on the microscopic image of skin with immunohistochemical staining for von Willebrand factor (vWF). A positive antigen-antibody reaction is seen as a tanbrown color. The staining in the NORM and STZ groups was observed only within the blood vessels, so the intensity of staining in the skin section was assessed as lack of staining (-). NORM – normoglycemic group; STZ – diabetic group; (epi) – epidermis; (hf) – hair follicle; (sg) – sebaceous gland; (fat) - subcutaneous tissue; (msc) – panniculus carnosus; (ves) – blood vessel; (-) lack of staining. Magnification ×200.

Figure S6. Effect of diabetes on the microscopic image of skin stained immunohistochemically for zonula occludens-1 (ZO-1). A positive antigen–antibody reaction appears as a tan-brown color. Weak staining (+) was observed in both the NORM and STZ groups within the epidermis, hair follicles, sebaceous glands, and blood vessels. NORM – normoglycemic group; STZ – diabetic group; (epi) – epidermis; (hf) – hair follicle; (sg) – sebaceous gland; (fat) – subcutaneous tissue; (msc) – panniculus carnosus; (ves) – blood vessel; (-) no color reaction. Magnification ×200.

Figure S7. Permeability of the human dermal microvascular endothelial cell (HMEC-1) monolayer under normal (NORM) or hyperglycemic (HG) conditions. **(a)** after 15 minutes of aldosterone exposure, and **(b)** after 60 minutes of aldosterone exposure. ALDO – aldosterone; VEH – aldosterone solvent. Results are presented as mean ± SD; n=6-9. Statistical relationships among groups were visualized using compact letter displays, where groups sharing the same letter are not significantly different, whereas groups without a common letter differ significantly ($p < 0.05$).
