# Supplementary figures and images for: The Role of Aldosterone in Vascular Permeability in Diabetes
Source: Cells. 2026 Jan 5;15(1):89. doi: 10.3390/cells15010089 (PMC12785615; doi:10.3390/cells15010089)

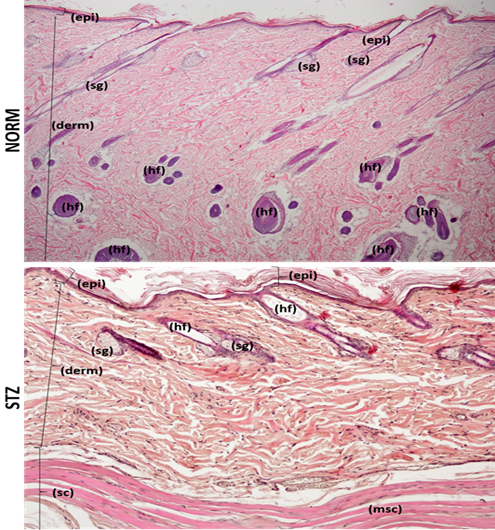

Supplement: Supplementary file 1 [file cells-15-00089-s001.zip › Figure S1.tif]

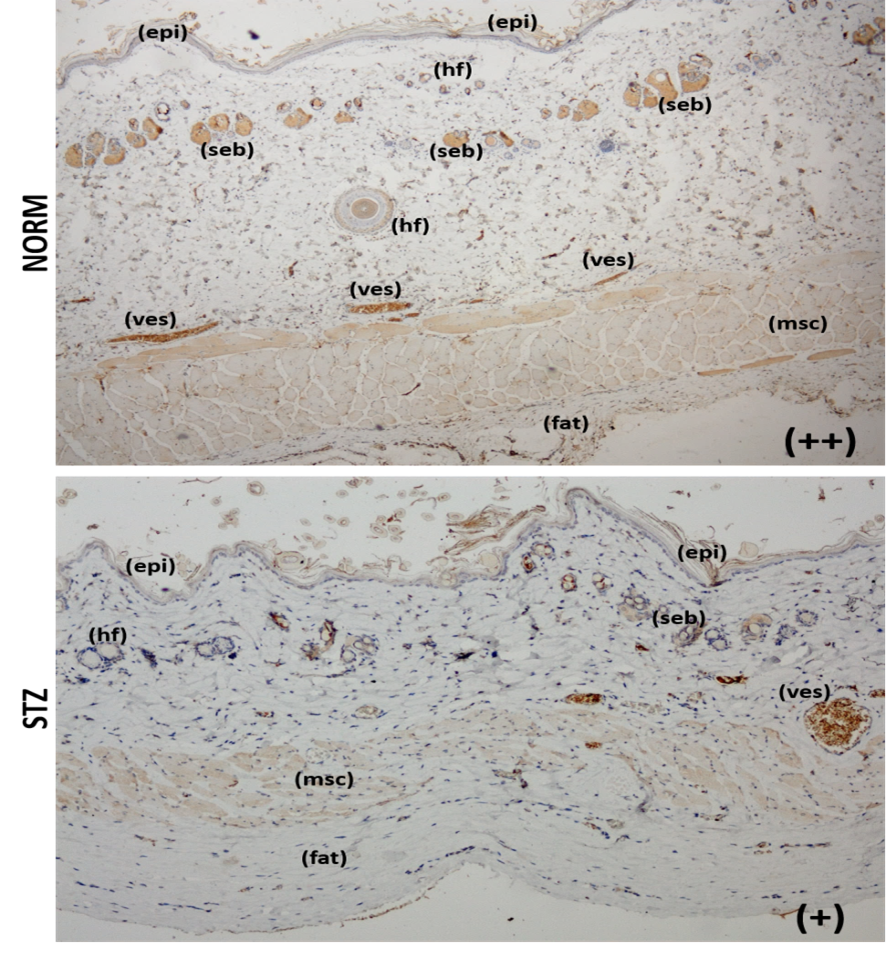

Supplement: Supplementary file 1 [file cells-15-00089-s001.zip › Figure S2.tif]

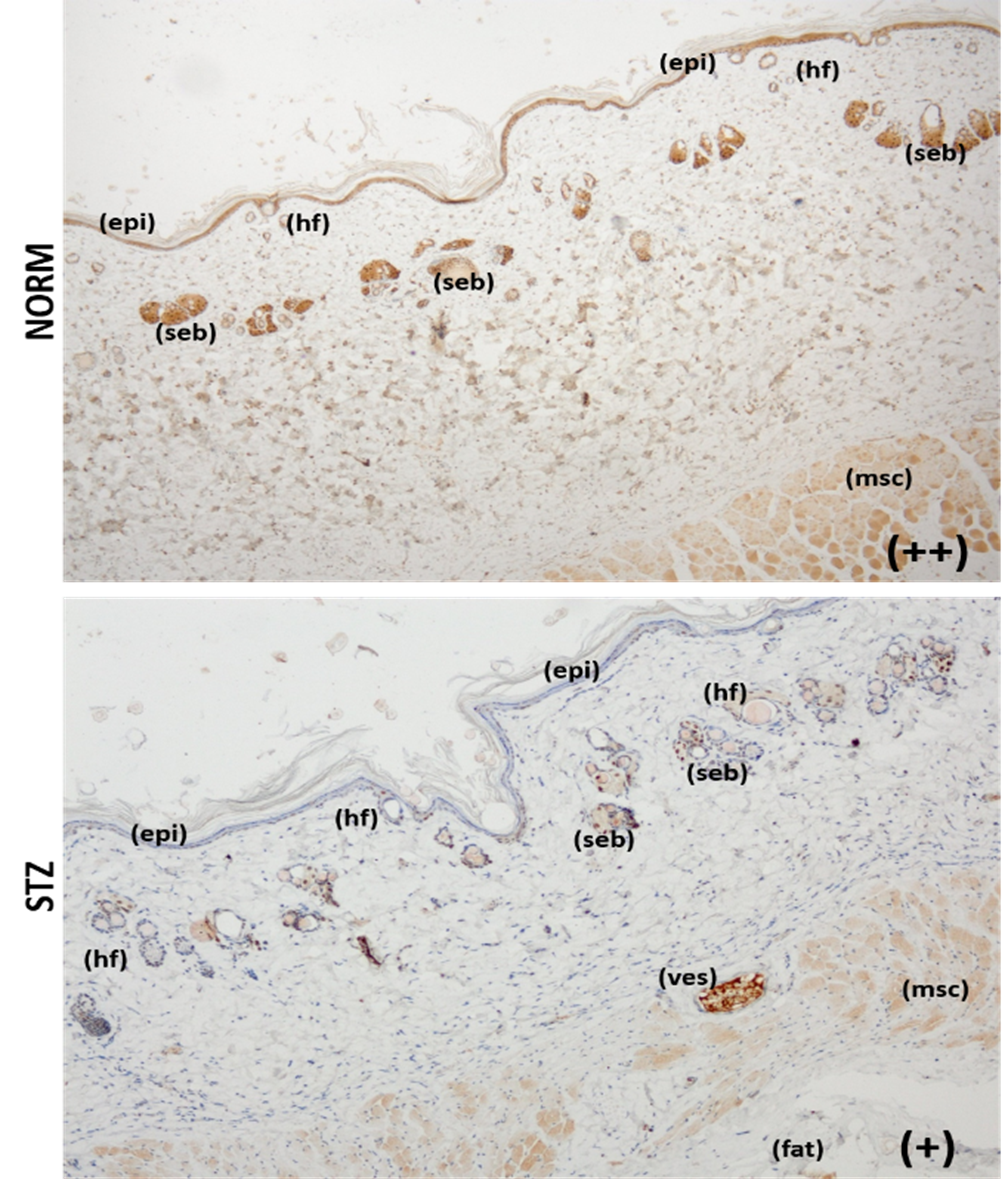

Supplement: Supplementary file 1 [file cells-15-00089-s001.zip › Figure S3.tif]

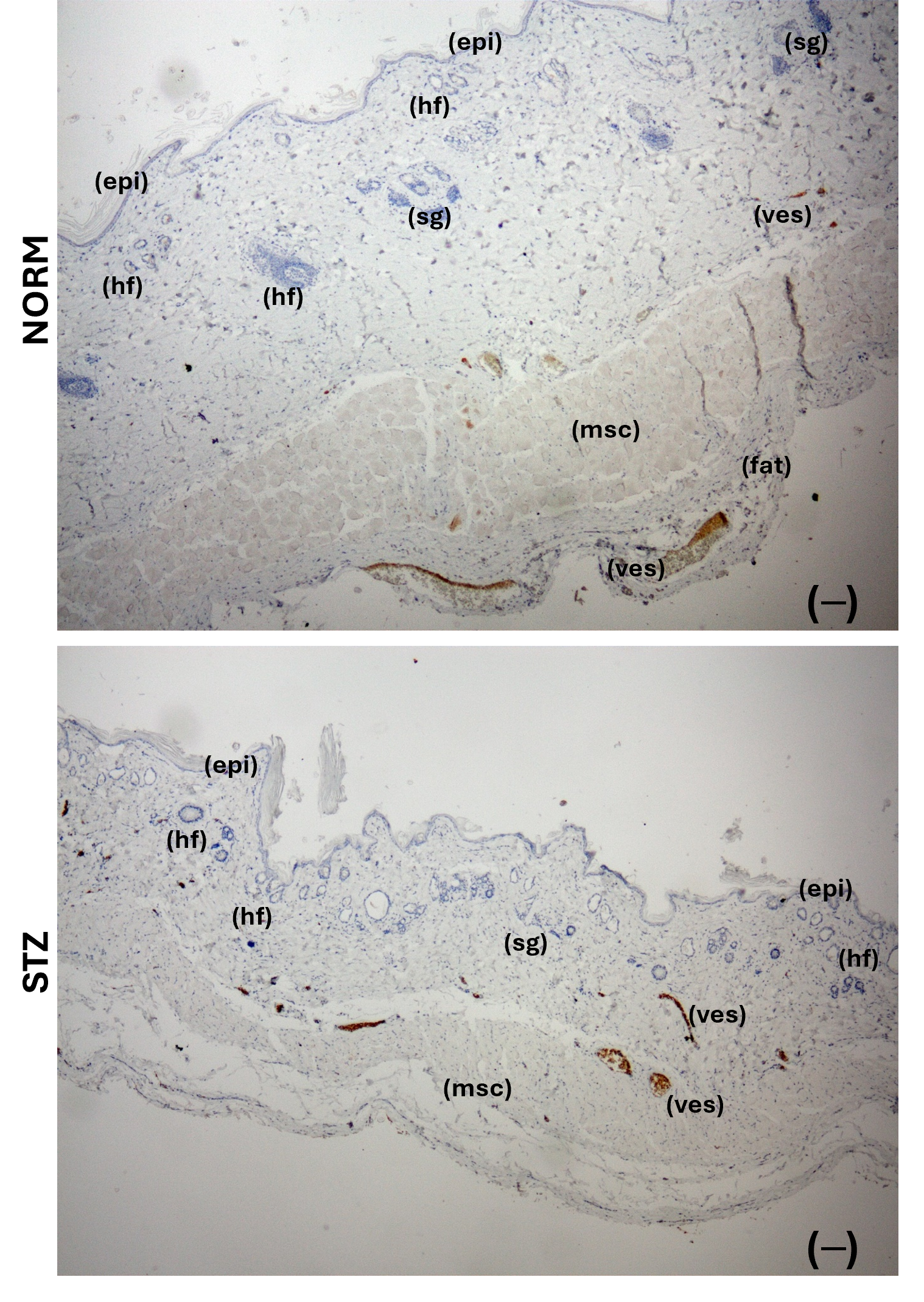

Supplement: Supplementary file 1 [file cells-15-00089-s001.zip › Figure S4.tif]

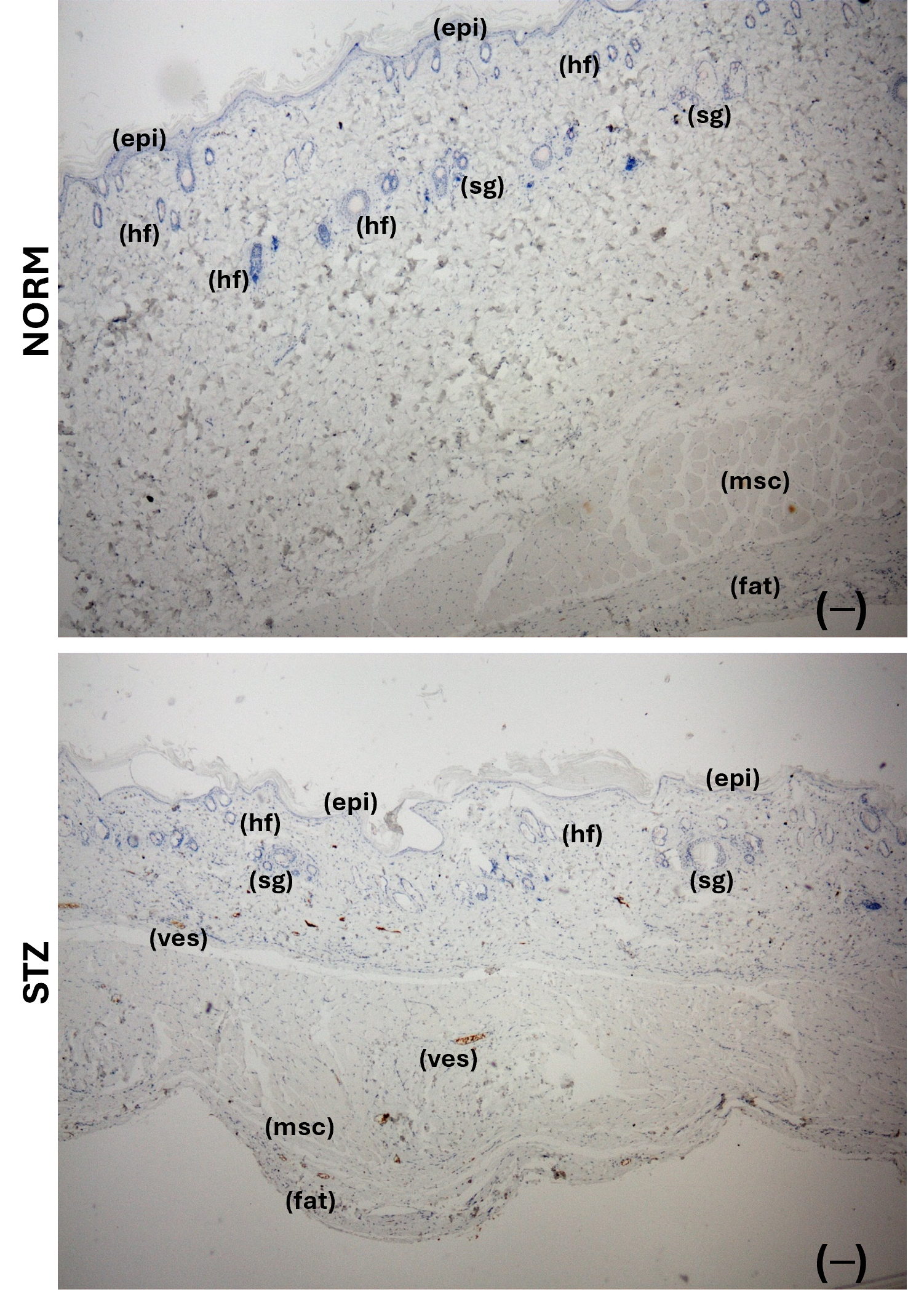

Supplement: Supplementary file 1 [file cells-15-00089-s001.zip › Figure S5.tif]

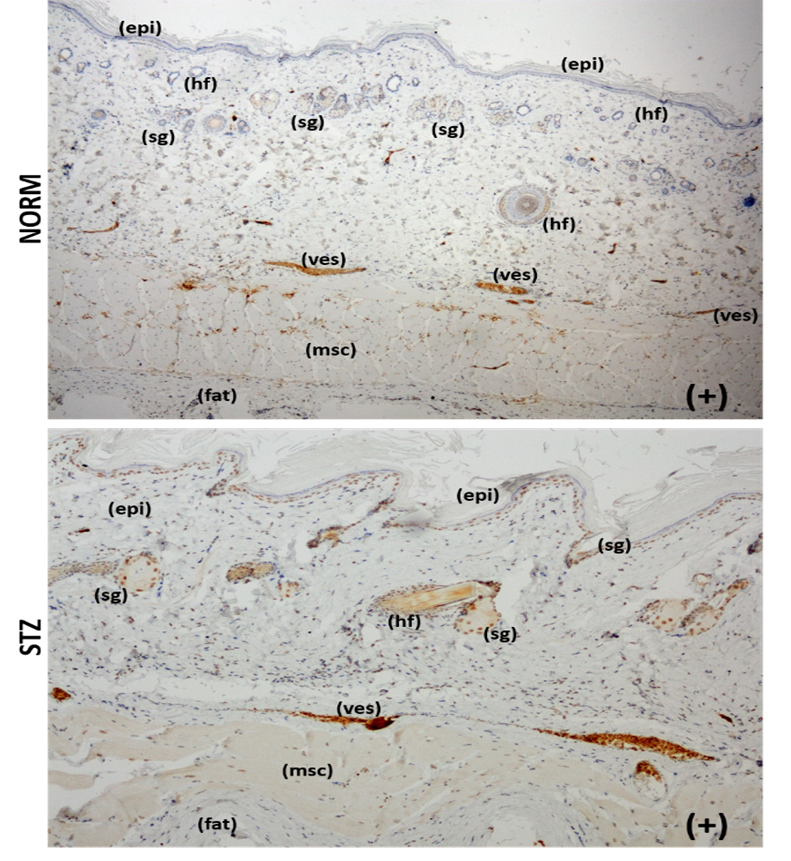

Supplement: Supplementary file 1 [file cells-15-00089-s001.zip › Figure S6.tif]

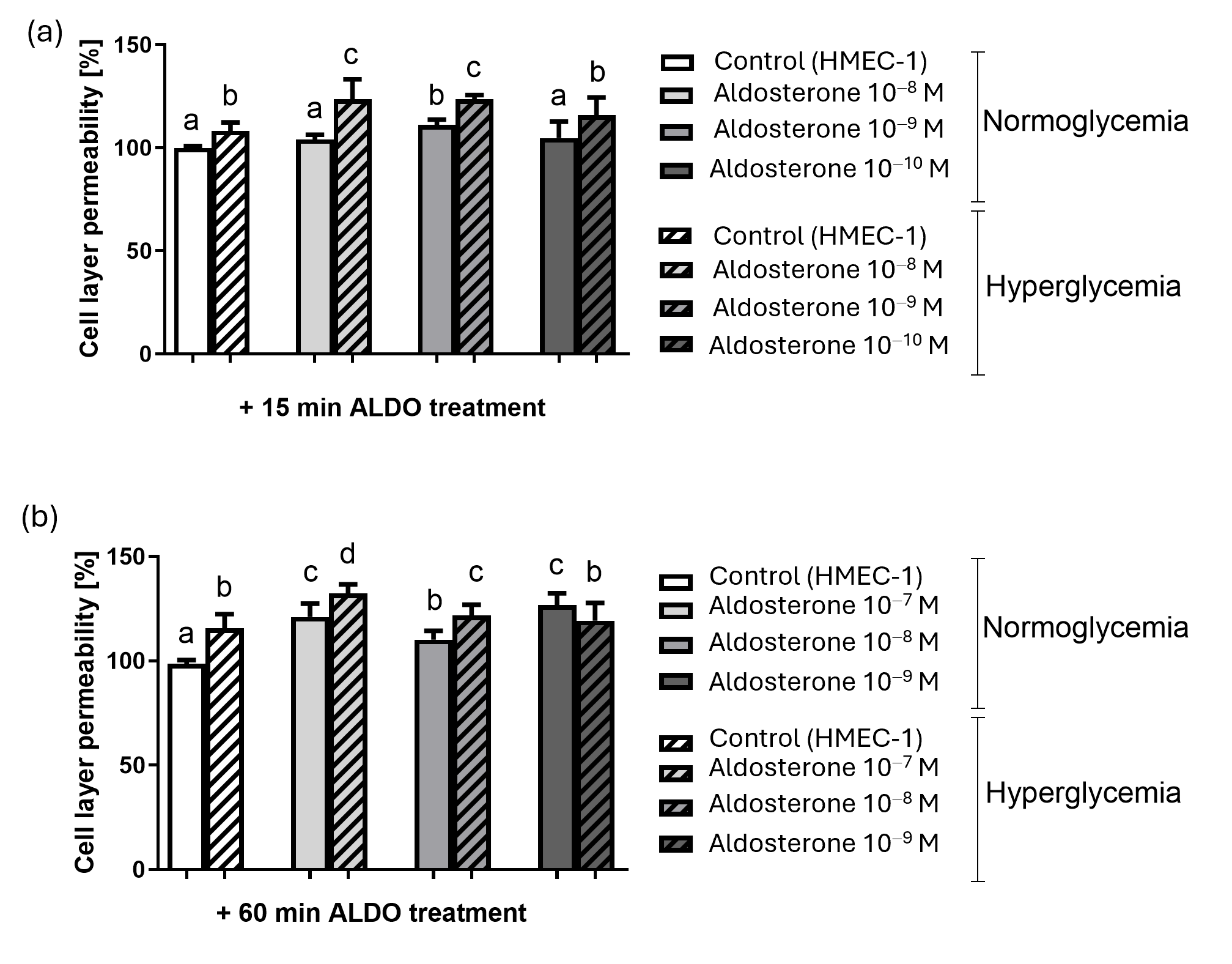

Supplement: Supplementary file 1 [file cells-15-00089-s001.zip › Figure S7.tif]
